# Sex differences in out-of-hospital cardiac arrest

**DOI:** 10.1093/ehjopen/oeaf047

**Published:** 2025-04-28

**Authors:** Nertila Zylyftari, Mads Wissenberg, Filip Gnesin, Amalie Lykkemark Møller, Elisabeth Helen Anna Mills, Sidsel G Møller, Britta Jensen, Kristian Bundgaard Ringgren, Hanno L Tan, Fredrik Folke, Gunnar Gislason, Christian Torp- Pedersen, Christina Ji-Young Lee

**Affiliations:** Department of Cardiology, Herlev and Gentofte Hospital, Copenhagen University Hospital, Gentofte Hospitalsvej 6, 2900 Hellerup, Denmark; Department of Cardiology, Nordsjællands Hospital, Dyrehavevej 29, 3400 Hillerød, Denmark; Department of Emergency Department, Svendborg Hospital, Baagøes Alle 15, 5700 Svendborg, Denmark; Department of Cardiology, Herlev and Gentofte Hospital, Copenhagen University Hospital, Gentofte Hospitalsvej 6, 2900 Hellerup, Denmark; Department of Cardiology, Nordsjællands Hospital, Dyrehavevej 29, 3400 Hillerød, Denmark; Department of Public Health, University of Copenhagen, Blegdamsvej 3B, 2200 Copenhagen, Denmark; Cancer Surveillance and Pharmacoepidemiology, Danish Cancer Society Research Center, Danish Cancer Society, Strandboulevarden 49, 2100 Copenhagen, Denmark; Department of Cardiology, Aalborg University Hospital, Hobrovej 18-22, 9000 Aalborg, Denmark; Department of Clinical Medicine, Aalborg University, Mølleparkvej 4, 9000 Aalborg, Denmark; Copenhagen Emergency Medical Services, Telegrafvej 5, 2750 Ballerup, Denmark; Public Health and Epidemiology, Department of Health Science and Technology, Aalborg University, Selma Lagerløfs Vej 249, 9260 Gistrup, Denmark; Department of Anesthesia and Intensive Care, Aalborg University Hospital, Hobrovej 18-22, 9000 Aalborg, Denmark; Department of Clinical and Experimental Cardiology, Amsterdam UMC, University of Amsterdam, De Boelelaan 1117, 1081 HV Amsterdam, The Netherlands; Department of Cardiology, Netherlands Heart Institute, Moreelsepark 1, 3511 EP Utrecht, The Netherlands; Department of Cardiology, Herlev and Gentofte Hospital, Copenhagen University Hospital, Gentofte Hospitalsvej 6, 2900 Hellerup, Denmark; Copenhagen Emergency Medical Services, Telegrafvej 5, 2750 Ballerup, Denmark; Department of Clinical Medicine, University of Copenhagen, Blegdamsvej 3B, 2200 Copenhagen, Denmark; Department of Cardiology, Herlev and Gentofte Hospital, Copenhagen University Hospital, Gentofte Hospitalsvej 6, 2900 Hellerup, Denmark; Department of Clinical Medicine, University of Copenhagen, Blegdamsvej 3B, 2200 Copenhagen, Denmark; The Danish Heart Foundation, Vognmagergade 7, 1120 Copenhagen, Denmark; Department of Cardiology, Nordsjællands Hospital, Dyrehavevej 29, 3400 Hillerød, Denmark; Department of Public Health, University of Copenhagen, Blegdamsvej 3B, 2200 Copenhagen, Denmark; Department of Cardiology, Nordsjællands Hospital, Dyrehavevej 29, 3400 Hillerød, Denmark; Copenhagen University Hospital—Steno Diabetes Center Copenhagen, Borgmester Ib Juuls Vej 83, Herlev Hospital, 2730 Herlev, Denmark

**Keywords:** Out-of-hospital cardiac arrest (OHCA), Women, Female sex, 30-day survival, ESCAPE-NET, PARQ-COST

## Abstract

**Aims:**

The impact of resuscitation care initiatives on sex-differences in out-of-hospital cardiac arrest (OHCA) survival remains unclear. We aim to examine sex-differences in characteristics and survival.

**Methods and results:**

This Danish register-based study (2001–2020) included adult patients with a presumed cardiac cause of arrest. Temporal trends in survival were stratified by sex and subgroups: (i) bystander-witnessed status; (ii) bystander cardiopulmonary resuscitation (CPR); (iii) initial shockable heart rhythm; and age groups of <50, 50–75, and >75 years. To examine the association between sex and survival, we conducted adjusted logistic regression analyses. Among 50 066 OHCAs, women represented 34%. Women were older, had more chronic obstructive pulmonary disease, and lower prevalence of cardiovascular and cardiometabolic conditions than men. Women also had more OHCA at home (83.4 vs. 74.1%), fewer witnessed arrests (48.1 vs. 52.9%), half the probability of initial shockable heart rhythm (13.6 vs. 27.6%), and similar rates of receiving bystander-CPR. Survival rates improved over time for both sexes, but men had higher 30-day survival than women, even in subgroup and adjusted analyses [odds ratios (OR): 1.29; 95% confidence intervals (CI): 1.15–1.45, *P* < 0.001]. Sex-differences in survival were larger among those aged 50–75 years (in absolute and relative rates) and bystander witnessed arrests, while differences were smaller in those with initial shockable heart rhythms.

**Conclusion:**

Despite increases in 30-day survival for both sexes, women consistently had lower survival rates than men. Sex-differences were larger among those aged 50–75 years or with bystander witnessed arrests, but smaller in the subset of patients with an initial shockable heart rhythm.

## Graphical Abstract

**Graphical Abstract:**
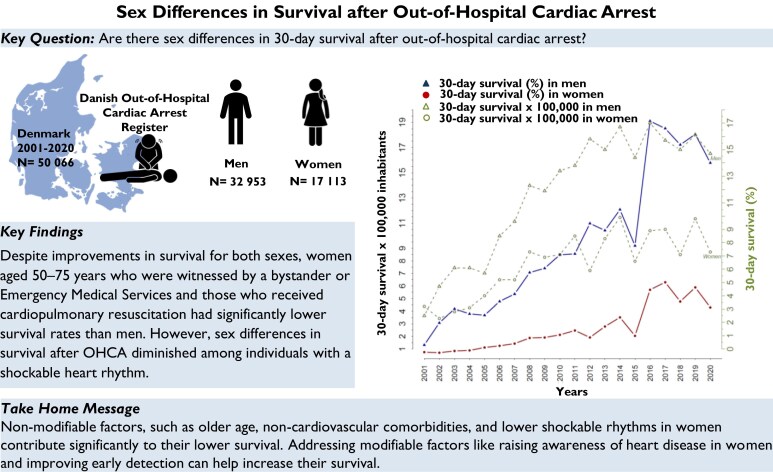

## Introduction

Over the past decades, ongoing initiatives have aimed to strengthen all links of the ‘chain of survival’, significantly improving cardiac arrest survival rates.^1–6^ Despite these efforts, out-of-hospital cardiac arrest (OHCA) accounts for 3.5 million deaths annually and represents half of all cardiac-related deaths worldwide,^7–11^ with a survival rate of <10%.^7,12^

Since 2001, major updates to resuscitation guidelines have been introduced.^13–16^ These guidelines emphasize early intervention, high-quality bystander cardiopulmonary resuscitation (CPR), defibrillation, and comprehensive post-resuscitation care. In Denmark, during the last two decades, there have been increased numbers of available automated external defibrillators, increased awareness of training the public in CPR, improved dispatcher recognition of OHCA, and, in 2017, the launch of a smartphone app enabling dispatch-assisted CPR by volunteer citizens.^4,17,18^ Consequently, from 2001 to 2020, OHCA survival rates have increased four-fold.^19,20^ Previous studies have examined the differences in survival rates between women and men with inconsistent findings.^21–31^ Women who experience OHCA are often older, have more comorbidities, and often experience non-ischaemic causes of cardiac arrest compared with men.^24,32^ They also have a lower incidence of initial shockable heart rhythm, are less witnessed, and receive less CPR, which all are shown to significantly decrease survival after OHCA.^22–24^

In Denmark, between 2001 and 2010, we found that women had lower survival rates than men.^24^ However, after 2010, Denmark has focused more on implementing a high-resuscitation practice.^20^ In 2020, bystander CPR increased to 80%, leading to an increase in 30-day survival from 10% in 2010 to 14% in 2020.^33^ Given the improvements in resuscitation practices, it is unclear whether sex differences in OHCA survival have changed over time. Using Denmark’s comprehensive national OHCA data, we conducted a 20-year nationwide study to explore sex differences in characteristics and 30-day survival rates among OHCA patients. We also performed multivariate analyses to examine the relationship between sex and OHCA survival, considering factors such as age, comorbidities, medical therapy, and cardiac arrest-related factors.

## Methods

### Study design and population

This retrospective cohort study utilized registry data to investigate patients in Denmark who experienced their first OHCA from 1 June 2001 to 31 December 2020, between 18 and 100 years of age, with a presumed cardiac cause of arrest and not witnessed by Emergency Medical Services (EMS) personnel. To define the presumed cardiac cause of OHCA we used diagnosis codes from death certificates and discharge diagnoses from all Danish hospitals. Events with diagnosis codes including cardiac disease, unknown disease, or unexpected collapse were defined as presumed cardiac cause of arrest.

Trauma including various accidents, violent attacks, and attempted suicide were together with drug overdose defined as external causes, regardless of other diagnoses.^15^ Because of fundamental differences in the underlying pathophysiology and system of care and survival,^34^ we wanted to examine a more homogenous group of patients throughout the 20-year study period. Therefore, this study focuses on OHCAs with presumed cardiac cause of arrest. Furthermore, the EMS-witnessed patients were excluded from the main analysis to ensure a more homogeneous study population, as this subgroup has higher survival rates.^35^ We conduct a separate analysis to explore sex differences specifically among EMS-witnessed with a presumed cardiac cause of arrests.

### Data sources

In Denmark, all residents have a unique civil registration number, enabling linkage between the nationwide Danish registries on an individual level.^36^ We identified all OHCA patients in Denmark (1 June 2001–31 December 2020) from the national register, Danish Cardiac Arrest Register.^36^ The data collected from 2001 was initially registered manually in paper forms, whereas from 2016, an electronic form of registration was nationally introduced in all ambulances and medical critical care units. This registry includes the date of OHCA, location (private or public), response time in minutes (the interval time from the call received to vehicle halt); witness status (bystander, EMS, or unwitnessed), bystander interventions of CPR or defibrillation, initial recorded heart rhythm (ventricular fibrillation, pulseless ventricular tachycardia, pulseless electrical activity, or asystole), return of spontaneous circulation (ROSC) at hospital arrival, as described elsewhere.^24^ The cardiac arrest-related factors were missing for 20.7%. However, no major differences were seen in patient and cardiac arrest-related characteristics for patients with missing data vs. those without (not shown). From the Danish National Patient Register, we retrieved information on comorbidities using discharge diagnosis codes according to the International Classification of Disease from the emergency department, inpatient, and outpatient hospital admissions.^37^ Data on in-hospital invasive procedures were also extracted from the Danish National Patient Register to outline pre-OHCA medical history up to 10 years before the event and post-arrest procedures conducted from the onset of cardiac arrest to 30 days thereafter, encompassing acute and subacute interventions. Information on all redeemed medical prescriptions was obtained from the Danish National Prescription Register with drugs classified according to the Anatomical Therapeutic Chemical system.^38^ Diagnosis codes up to 10 years before OHCA were used to define comorbidities, and the redeemed prescriptions within 180 days before OHCA were used to define the patients’ therapy in our baseline table.

Demographic information including age, sex, and vital status was obtained from the Danish Civil Registration System.^39^ Information on causes of death was obtained from death certificates from the National Causes of Death Register.^40^

### Outcomes

The primary endpoints of the study were to explore patient characteristics and 30-day survival according to sex, presented as (i) absolute numbers per 100 000 inhabitants per year (based on the Danish population), and (ii) relative survival rates in percentages per year (based on OHCA population). Reporting both measurements helps to minimize bias related to the misclassification of cardiac arrests. Absolute measurements (*n* per 100 000 inhabitants) are not sensitive to misclassification but sensitive to the underlying risk of cardiac arrest in a subgroup. Relative survival has the potential to be biased as survival is critically influenced by whether a collapse is considered sudden death or cardiac arrest, as also shown previously.^41^

### Statistics

Descriptive statistics stratified by sex were used to summarize categorical variables and medians with inter-quartile ranges (IQR) for continuous variables. We performed a temporal trend analysis of 30-day survival according to sex over 20 years. Absolute numbers of OHCA incidence and survivors per 100 000 inhabitants were calculated based on the Danish population by sex in the same age group as included in the Danish Cardiac Arrest Register.

Hereafter, we examined patient characteristics and temporal trends of 30-day survival divided into the following dichotomic subgroups: (i) shockable/non-shockable rhythm; (ii) witnessed/non-witnessed by a bystander; (i) received bystander CPR/no bystander CPR; and age groups of <50, 50–75-, and >75-years. The latter cut-offs were chosen based on the age distribution in the population and clinical relevance.

In supplementary analyses, we examined sex differences in characteristics and survival outcomes in two distinct subgroups: (i) EMS-witnessed patients, who have higher survival rate, and (2) patients fulfilling the Utstein comparator definition (non-traumatic, bystander-witnessed OHCA with an initial shockable heart rhythm). The Utstein comparator group is commonly used to compare resuscitation performance across EMS systems and represents the OHCA population with the highest potential to benefit from resuscitative efforts.^42^

Lastly, we performed a logistic regression analysis to estimate the association of OHCA survival with sex, adjusting for age groups, comorbidities, medication use, cardiac arrest–related factors, and invasive procedures on the day of OHCA. The model was created by considering different variables identified as possible confounders using a Directed Acyclic Graph based on expert knowledge. All variables were included in the initial model under the assumption that missing data were not missing at random. Consequently, observations with missing data were excluded from the logistic regression analyses, hence we conducted a complete case analysis. We reported Nagelkerke’s *R*² as a measure of model fit, and odds ratios (OR) with 95% CI and two-sided *P*-values, using a significance level of 5%. We also tested for interaction between sex and (i) initial shockable heart rhythm, (ii) ischaemic heart disease (IHD), and (iii) age. The analysis included data from the second decade, as analyses before 2010 are presented elsewhere.^24^ Data management and statistical analyses were performed with R version 4.4.1.^43^

### Ethics

This study was approved by the Danish Data Protection in Denmark, and registry-based studies do not require ethical approval. However, the information on the study population was encrypted and rendered anonymous by Statistics Denmark.

## Results

### Patient characteristics

Among 50 066 OHCAs included in the study (see Supplementary material online, *Figure S1*), around 34% were women. Compared to men, they were older and had lower socioeconomic status, a higher burden of chronic obstructive pulmonary disease and psychiatric diseases, along with more redeemed prescriptions for antibiotics and QT-prolonging drugs (*Table 1*). Conversely, cardiovascular and cardiometabolic comorbidities were less prevalent in women. Regarding cardiac arrest-related factors, women more often suffered OHCA in a private home setting, had less witnessed arrest, were less likely to receive defibrillation, and were nearly half as likely to register with initial shockable heart rhythm (*Table 1*). There were no sex differences in patients receiving bystander CPR.

**Table 1:** Baseline characteristics

Characteristics	Women (*n* = 17 113)	Men (*n* = 32 953)	Total (*n* = 50 066)
Age (years) (median, IQR)	76 [66, 84]	71 [61, 80]	73 [63, 81]
Education levels			
Basic education	10 962 (64.1)	15 038 (45.6)	26 000 (51.9)
General or vocational upper secondary	4366 (25.5)	13 095 (39.7)	17 461(34.9)
Bachelor, Master, or Doctoral Degree or equivalent	1785(10.4)	4820 (14.6)	6605 (13.2)
Civil status			
Married	6169 (36.0)	19 050 (57.8)	25 219 (50.4)
Widow	6081 (35.5)	3675 (11.2)	9756 (19.5)
Number of persons at home			
One (living alone)	8766 (51.2)	9424 (28.6)	18 190 (36,3)
Two	6368 (37.2)	18 046 (54.8)	24 414 (48.8)
Household income levels			
Low	4327 (25.3)	7287 (22.1)	11 614 (23.2)
Medium	9,33 (54.5)	15 908 (48.3)	25 241 (50.4)
High	3453 (20.2)	9758 (29.6)	13 211 (26.4)
Comorbidities			
Hypertension	9201 (53.8)	18 313 (55.6)	27 514 (55.0)
Type 2 diabetes	2460 (14.4)	5457 (16.6)	7917 (15.8)
Myocardial infarction	1356 (7.9)	3547 (10.8)	4903 (9.8)
Ischaemic heart disease	2873 (16.8)	8011 (24.3)	10 884 (21.7)
Heart failure	2823 (16.5)	6563 (19.9)	9386 (18.7)
Atrial fibrillation	2912 (17.0)	6314 (19.2)	9226 (18.4)
Stroke	1811 (10.6)	3566 (10.8)	5377 (10.7)
Chronic obstructive pulmonary disease (COPD)	3284 (19.2)	4552 (13.8)	7836 (15.7)
Neurological diseases	3698 (21.6)	5717 (17.3)	9415 (18.8)
Psychiatric disorders	2200 (12.9)	2471 (7.5)	4671 (9.3)
Drug or alcohol abuse 180 days before OHCA	1366 (8.0)	3154 (9.6)	4520 (9.0)
Pre-arrest invasive in-hospital procedures (10 years before OHCA)			
Coronary artery bypass graft surgery (CABG)	172 (1.0)	1110 (3.4)	1282 (2.6)
Percutaneous coronary intervention (PCI)	623 (3.6)	2210 (6.7)	2833 (5.7)
Pacemaker implantation	699 (4.1)	1720 (5.2)	2419 (4.8)
Coronary angiography (CAG)	1787 (10.4)	5535 (16.8)	7322 (14.6)
Surgery for cardiac arrhythmias or conduction disorders, including implantable cardioverter-defibrillator and radiofrequency ablation	169 (1.0)	795 (2.4)	964 (1.9)
Post-arrest invasive procedures (up to 30 days after OHCA)			
PCI the day of OHCA	379 (2.2)	1954 (5.9)	2333 (4.7)
PCI up to 30 days after OHCA	451 (2.6)	2268 (6.9)	2719 (5.4)
CAG	1106 (6.5)	4527 (13.7)	5633 (11.3)
Surgery for cardiac arrhythmias or conduction disorders, including ICD implantation and radiofrequency ablation -	372 (1.9)	1715 (4.6)	2087 (4.2)
Medication 180 days before and at the time of OHCA			
Beta blocker, calcium antagonist, or digoxin	7387 (43.2)	13 879 (42.1)	21 266 (42.5)
Antidepressant or anti-psychotic drugs	5611 (32.8)	6172 (18.7)	11 783 (23.5)
Anticoagulant drugs	2283 (13.3)	5525 (16.8)	7808 (15.6)
Systemic steroids	3675 (21.5)	4965 (15.1)	8640 (17.3)
COPD medication	4428 (25.9)	6157 (18.7)	10 585 (21.1)
Antianginal drugs (ivabradine, nitrate, nicorandil, and nitroglycerine)	1483 (8.7)	3218 (9.8)	4701 (9.4)
Statins	4327 (25.3)	10 399 (31.6)	14 726 (29.4)
Antibiotics 30 days before and at the time of OHCA	3488 (20.4)	4516 (13.7)	8004 (16.0)
QT-prolonging drugs 30 days before and at the time of OHCA	2418 (14.1)	2891 (8.8)	5309 (10.6)
Cardiac arrest related factors			
Arrest in private home	11 530 (83.4)	19 148 (74.1)	30 678 (77.3)
Missing (%)	3287 (19.2)	7103 (21.6)	10 390 (20.7)
Witnessed arrest	8175 (48.1)	17 280 (52.9)	25 445 (51.3)
Missing (%)	134 (0.7)	270 (0.8)	404 (0.8)
Cardiopulmonary resuscitation before ambulance arrival	9859 (58.0)	19 050 (58.1)	28 909 (58.1)
Missing (%)	114 (0.6)	192 (0.5)	306 (0.6)
Defibrillation before ambulance arrival	591 (3.6)	1962 (6.1)	2553 (5.2)
Missing (%)	501 (2.9)	855 (2.5)	1356 (2.7)
Initial shockable heart rhythm	2214 (13.6)	8696 (27.6)	10 910 (22.8)
Missing (%)	866 (5.0)	1440 (4.3)	2306 (4.6)
Median response time in minutes^a^ (IQR)	9 [6, 14]	10 [6, 15]	10 [6, 15]
Missing (%)	1453 (9.0)	2657 (8.9)	2657 (8.9)
Patient has return of spontaneous circulation (ROSC) or has Glasgow Coma Scale (GCS) > 8 at the hospital arrival	2972 (18.1)	6820 (21.5)	9792 (20.3)
Missing (%)	676 (3.9)	1178 (3.5)	1854 (3.6)

OHCA, out-of-hospital cardiac arrest; ICD, implantable cardioverter defibrillator; COPD, chronic obstructive pulmonary disease, GCS, Glasgow Coma Scale.

^a^Interval time from the recognition of OHCA to the first rhythm analysis by EMS.

#### The incidence of out-of-hospital cardiac arrest by sex

The OHCA incidence per 100 000 inhabitants increased modestly between 2001 and 2015, whereas in 2015–2016, the numbers increased drastically for both sexes coinciding with the transition to electronic data collection (see Supplementary material online, *Figure S2*). However, women had lower OHCA incidence compared to men throughout the whole study period.

### The 30-day survival rate of out-of-hospital cardiac arrest by sex

**Figure 1:**
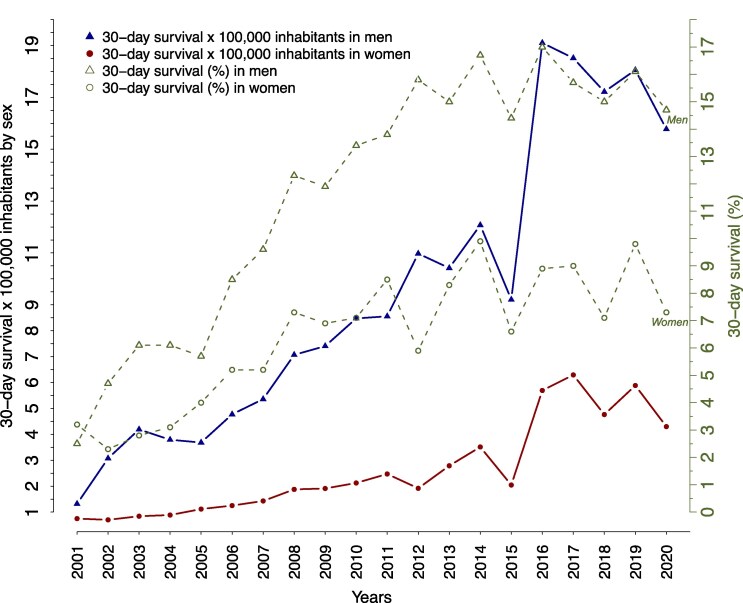
The crude 30-day out-of-hospital cardiac arrest survival by sex in percentage and number of survivors per 100 000 inhabitants per year.

Of all the OHCAs, 10.7% of OHCA patients survived, where men compared to women had higher survival rates (7 vs. 12.7%) and a higher absolute number of survivors (see Supplementary material online, *Figure S3*). The temporal trends of 30-day survival percentages increased significantly throughout the study period (*Figure 1*), and average percentages nearly doubled for both sexes from the first decade (2001–2010) to the second (2011–2020), reaching 4.7–8.1% for women and 8.1–15.4% for men, respectively. Similarly, the number of survivors significantly increased in both sexes but was lower for women, with lower incidence rates.

#### Stratification by age

Patient characteristics of women and men in each age category (<50, 50–75, >75 years) were consistent with those observed in the overall study population (see Supplementary material online, *Table S1*). However, among OHCA patients <50 years, sex differences in cardiac and cardiometabolic comorbidities were less pronounced than those over 50 years. Regarding cardiac arrest factors, among those aged 50–75 years, women more often had their OHCA at a private home (84.0 vs. 71.4%) and were less witnessed than men when compared to the other two age groups (<50: 49.9 vs. 50.3%; 50–75: 46.0 vs. 53.8%; >75: 49.6 vs. 52.1%). The overall rate of initial shockable heart rhythm was much lower in women compared to men (<50: 28.9 vs. 34.1%; 50–75: 15.5 vs. 31.8%; >75: 9.9 vs. 19.9%). This difference was less pronounced in women <50 years compared to the other two age groups. Return of spontaneous circulation upon hospital arrival rates were lower in women aged 50–75 years and >75 years while higher in women aged <50.

**Figure 2:**
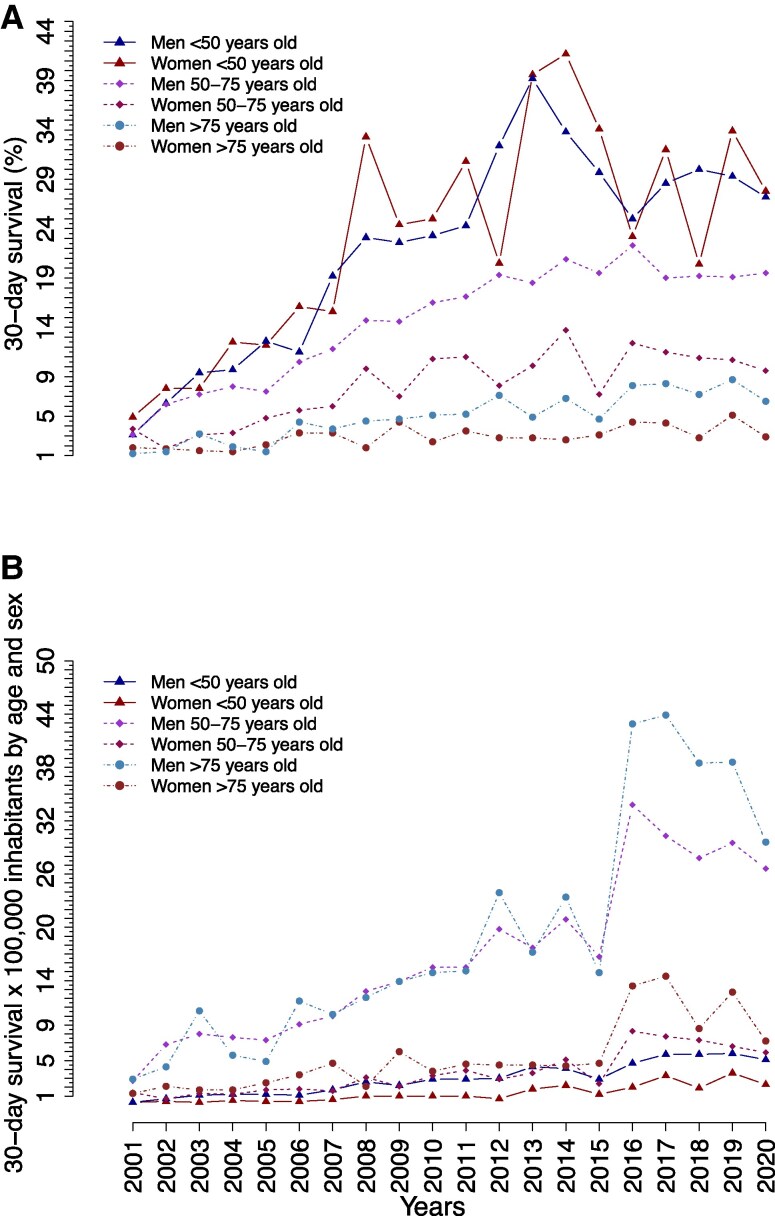
The crude 30-day out-of-hospital cardiac arrest survival according to sex and age categories (<50, 50–75, and >75 years old) shown in (*A*) percentages and (*B*) number of survivors per 100 000 inhabitants per year.

*Figure 2* shows the 30-day survival between 2001 and 2020 stratified by sex and age categories (<50, 50–75, and >75 years) in relative (*Figure 2A*) and absolute measurements (*Figure 2B*). Survival rates were highest for patients aged <50 years, with no sex differences over time. The survival rates were lower for patients over 50 years, whereas for those >75 years, women had nearly half of the chances of survival compared to men (women: 2.9% vs. men: 6.5% in 2020). Sex differences in survival were highest among patients aged 50–75 years (women: 9.6% vs. men: 19.5% in 2020), which appeared to be persistent over time when presented in both percentages (*Figure 2A*), and numbers of survivors ×100 000 inhabitants by age and sex (*Figure 2B*).

#### Stratification by cardiac arrest factors

Patient characteristics for both sexes, when stratified by witnessed status and bystander CPR provision were consistent with the baseline characteristics of the main analysis (see Supplementary material online, *Tables S2* and *S3*). While, among those with initial shockable heart rhythm, women were younger, received less bystander CPR, and had higher chances of achieving ROSC at hospital arrival compared to men (see Supplementary material online, *Table S4*).

**Figure 3:**
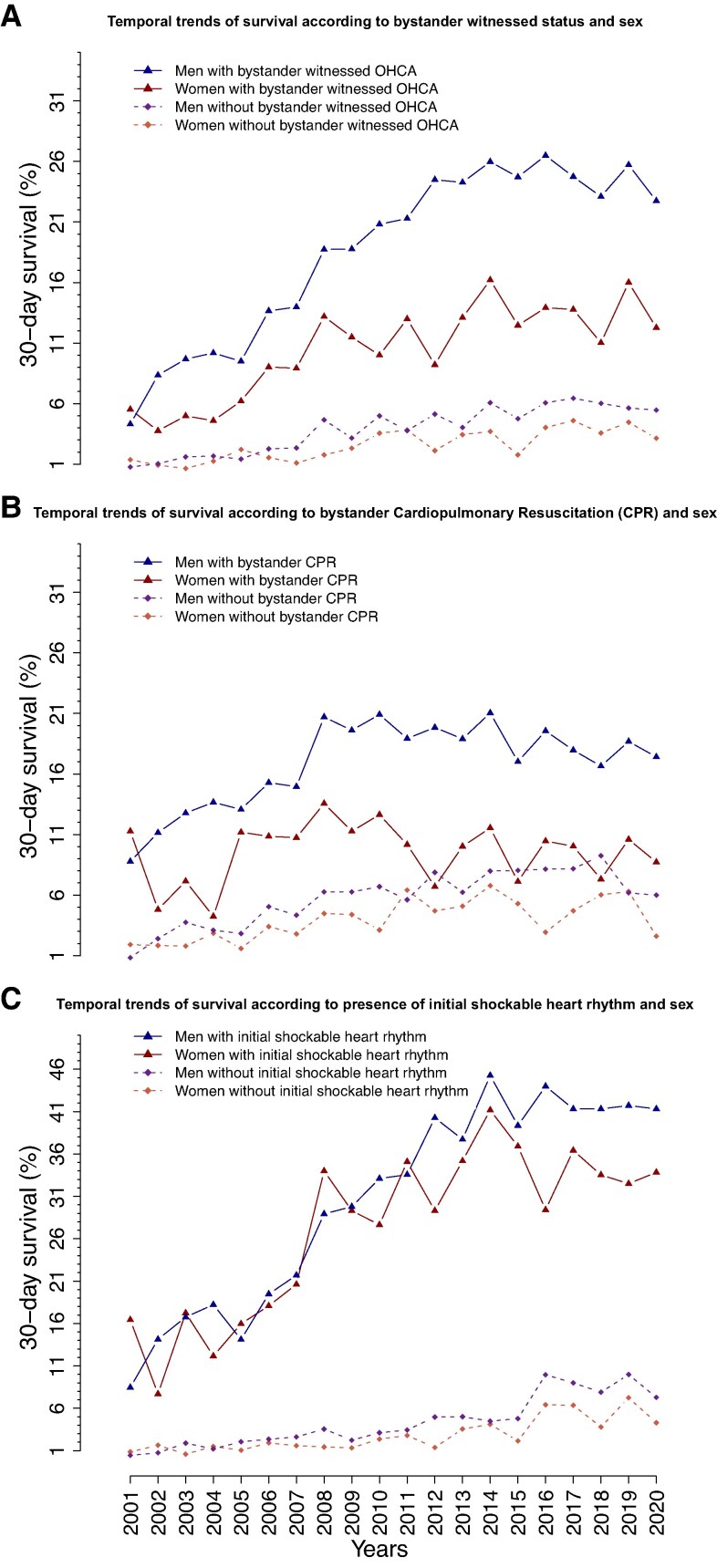
The crude 30-day survival rates of out-of-hospital cardiac arrest patients according to sex and (*A*) bystander-witnessed status, (*B*) cardiopulmonary resuscitation efforts from bystander, and (*C*) first recorded heart rhythm.

In the subgroup analysis of temporal trends, men had higher survival rates than women, regardless of whether OHCA was witnessed, whether they received bystander CPR, or whether they had an initial shockable or non-shockable heart rhythm. Sex differences in survival were more pronounced in witnessed cases and those who received bystander CPR. In contrast, sex differences in survival among patients with shockable rhythm were smaller (*Figure 3*). Temporal trend analysis of these cardiac arrest-related factors showed a marked increase in bystander CPR for both sexes. While rates of witnessed arrests showed steady rates with sex differences (see Supplementary material online, *Figure S4*). In contrast, shockable heart rhythm decreased over time with consistent sex differences.

#### The emergency medical services-witnessed OHCAs and Utstein comparator group

In the EMS-witnessed and Utstein comparator group, patient characteristics according to sex were similar to the baseline table, however, sex differences were less pronounced (see Supplementary material online, *Tables S5* and *S6*). In the Utstein comparator group, women were younger, had more cardiac comorbidities, experienced OHCA more at home, received less bystander CPR, and had a higher chance of achieving ROSC upon hospital arrival compared to men (see Supplementary material online, *Table S6*).

The overall 30-day survival rate among OHCAs witnessed by EMS and the Utstein comparator group increased in both sexes over time, and the rates were as expected higher compared to the main analysis (see Supplementary material online, *Figures S5* and S6). Among EMS-witnessed OHCAs, there was a marked survival difference among men and women (see Supplementary material online, *Figure S5*). In the Utstein comparator group, the sex difference in survival was initially smaller, but it increased after 2015, with women having lower survival rates than men (see Supplementary material online, *Figure S6*).

### Association between patient characteristics and 30-day survival of OHCA

**Figure 4:**
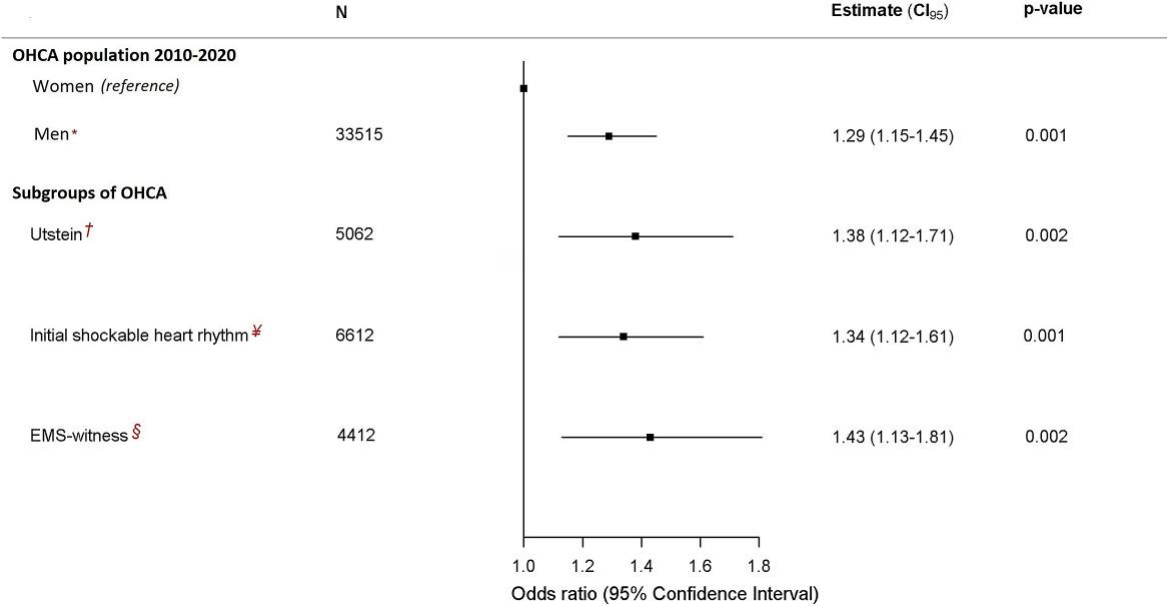
Association between sex and 30-day survival following out-of-hospital cardiac arrest in adjusted analyses. Odds ratio of 30-day survival by sex and in subgroups of out-of-hospital cardiac arrest population in men as compared to women (the reference group). EMS, Emergency Medical Services; OHCA, out-of-hospital cardiac arrest. *R*^2^ = 0523. The model with the main population (*) was adjusted for age, comorbidities (respiratory diseases, diabetes type 2, ischaemic heart disease, heart failure, and hypertension), medication (antidepressant and psychotropic drugs, and short therapy within 30 days to out-of-hospital cardiac arrest with antibiotics, QT-prolonging drugs), cardiac related factors (location of arrest, witnessed status, initial shockable heart rhythm, bystander defibrillation, bystander cardiopulmonary resuscitation, response time, and return of spontaneous circulation upon hospital arrival), and invasive procedure (PCI) on the day of out-of-hospital cardiac arrest. **^†^**Adjusted for age, comorbidities (respiratory diseases, diabetes type 2, ischaemic heart disease, heart failure, and hypertension), medication (antidepressant and psychotropic drugs, and short therapy within 30 days to out-of-hospital cardiac arrest with antibiotics, QT-prolonging drugs), cardiac related factors (location of arrest, defibrillation status, bystander cardiopulmonary resuscitation, response time, return of spontaneous circulation upon hospital arrival), and invasive procedure (PCI) on the day of out-of-hospital cardiac arrest. **^¥^**Adjusted for age, comorbidities (respiratory diseases, diabetes type 2, ischaemic heart disease, heart failure, and hypertension), medication (antidepressant and psychotropic drugs, and short therapy within 30 days to out-of-hospital cardiac arrest with antibiotics, QT-prolonging drugs), cardiac-related factors (location of arrest, witnessed status, defibrillation status, bystander cardiopulmonary resuscitation, response time, and the return of spontaneous circulation upon hospital arrival), and invasive procedure (PCI) on the day of out-of-hospital cardiac arrest. **^§^**Adjusted for age, comorbidities (respiratory diseases, diabetes type 2, ischaemic heart disease, heart failure, and hypertension), medication (antidepressant and psychotropic drugs, and short therapy within 30 days to out-of-hospital cardiac arrest with antibiotics, QT-prolonging drugs), cardiac-related factors (location of arrest, return of spontaneous circulation upon hospital arrival), and invasive procedure (PCI) on the day of out-of-hospital cardiac arrest.

In adjusted logistic regression analysis, the odds ratio of 30-day survival after OHCA was higher for men than women [OR 1.29; 95% confidence intervals (CI): 1.15–1.45, *P* < 0.001] (*Figure 4*). Results were consistent in subgroup analyses of the Utstein comparator group, witnessed by EMS personnel, having an initial shockable heart rhythm (*Figure 4*), and those with non-shockable heart rhythm (OR 1.26 CI: 1.08;1.47, *P* = 0.0029). While, in the subgroup of patients aged <50 years old, no differences in survival between sexes were found when comparing men to women (OR 0.82; 95% CI: 0.61;1.11, *P* = 0.2046). In a separate table are presented the ORs and CI for all other potential factors included in the logistic regression model (see Supplementary material online, *Table S7*). We observed a significant interaction between sex and the factors: age, ischaemicIHD, and initial shockable rhythm.

## Discussion

This nationwide study explored sex differences in characteristics and 30-day survival of patients with OHCA over 20 years. Women were older than men, had more non-cardiovascular comorbidities, and non-favourable cardiac arrest-related factors. Despite improvements in 30-day survival for both sexes over two decades, women consistently had lower survival rates than men. Sex differences in survival were the largest among patients aged 50–75 years compared to other age groups, while no significant difference was observed among those under 50 years. Sex differences in survival persisted also among OHCA patients witnessed by bystanders or EMS and those receiving CPR. However, smaller survival disparities were observed in patients with initial shockable heart rhythm and the Utstein comparator group.

### Patient characteristics

Consistently to previous literature, women in our cohort were older and more often presented more severe comorbidities.^24,29,31,32,44,45^ They experienced more arrests in private locations, and less frequently had shockable heart rhythms—factors linked to a lower survival.^3,4,22^ In contrast, men had more cardiac comorbidities and higher rates of shockable heart rhythms, which is associated with higher chances of survival.^46^ Even after adjusting for these factors, the 30-day survival among women remained lower compared to men.

Age is another key predictor of survival.^47^ In relation to this, the largest sex survival difference was observed among those aged 50–75 years, in both relative (percentage) and absolute (numbers ×100 000 inhabitants) measurements. An explanation of this finding might be due to sex differences in patient characteristics being the largest in the 50–75 years group, which determine the differences in survival. Older women often have a lower ‘physiological reserve’,^48^ which is reflected also in our cohort, where they had a lower percentage of initial shockable heart rhythms. Additionally, older women typically experience a higher burden of chronic diseases, and the combined effects of aging and declining oestrogen levels in middle-aged women may impact both the likelihood of a shockable heart rhythm and survival after cardiac arrest. Another explanation could be because of the delay in OHCA recognition and access to CPR, as women aged 50–75 years tend to live more alone at home than men within the same group, which makes it hard to improve early recognition and early intervention in case of cardiac arrest. Age is a non-modifiable factor, which makes it difficult to prevent OHCA in this subgroup. However, efforts could focus on raising awareness about the importance of seeking prompt healthcare in case of early symptoms before arrest, or e.g. developing devices for automatic detection and alerting in case of OHCA.^49^ Further, we observed no significant sex differences in survival among those under 50 years, possibly due to the lower burden of chronic comorbidities. Other studies have also pointed potential hormonal protective effects in younger women.^50^ Yet, further research is needed to clarify the role of oestrogen in survival after OHCA, given the conflicting results that been reported in this area.^51,52^

### Cardiac arrest factors

The lower survival in women compared to men, is consistent with some previous research.^22–25,27–30^ However, other studies have shown a higher survival among women,^21,53,54^ or no sex difference in survival after OHCA.^26,31^ These different results could be explained by the heterogeneity of studies in the field,^55^ as they differ in patient selection, risk factors, inclusion criteria, and endpoints, with some having endpoint survival to hospital discharge and favourable neurological outcome after OHCA,^53,56^ rather than 30-day survival. Furthermore, some studies had no information on cardiac-related factors, previous medical therapy, or comorbidities of patients included in the study,^26,53,57^ thus not being able to adjust for such factors.

We have previously shown that the temporal increase in 30-day crude survival between 2001 and 2010 was more marked in men compared to women, though women with shockable heart rhythms had better survival in adjusted analysis.^24^ The current study confirms lower survival in women during the 20-year study period, though the sex difference in survival persisted in adjusted analysis (2010–2020). Despite these disparities, survival rates have improved for both sexes over time, which differs from other studies. For example, a study from the Netherlands with similar findings did not show an increase in survival for both sexes.^29^ This discrepancy could be a result of the large awareness of resuscitative efforts in Denmark with several national initiatives taken to increase public awareness, leading to an increase in bystander CPR rates without sex difference over the years (see Supplementary material online, *Figure S4*). Whereas in the Netherlands, despite national efforts, women generally received less CPR than men.^23^

The reduced sex difference in survival rate among younger patients, those with shockable heart rhythm, and the Utstein comparator group could indicate that the differences between sexes in survival are mainly related to non-modifiable factors. The latter includes age and comorbidities, including a higher burden of non-cardiovascular disease, probably reflecting that women had a lower ‘physiological reserve’ with a high risk of non-shockable heart rhythm in case of arrest.^58^ Even among patients whose cardiac arrest was witnessed by bystanders or EMS and had initial shockable heart rhythm, differences in survival between sexes persisted. This finding highlight that time from collapse to recognition of arrest cannot explain the sex differences in survival. Additionally, when focusing on a more uniform subgroup, such as the Utstein comparator group characterized by the most favourable cardiac arrest circumstances, a smaller but still significant sex difference in survival remained evident. One explanation could be sex disparities in arrest aetiology and subsequent post-resuscitation care. For example, women in our cohort received fewer post-arrest interventions such as coronary angiography (CAG) and percutaneous coronary intervention (PCI), as also showed in the past.^32,55,59,60^ In multivariable analysis, the survival remained lower for women after adjusting for hospital treatments (PCI on the day of OHCA).

### Limitations

Firstly, we did not have clinical details to quantify the clinical status of the patients, including the severity of the comorbidities between men and women, including parameters on left ventricular functioning, or the exact cause of cardiac arrest, all of which could influence post-arrest management and survival outcomes. Nonetheless, the Danish National Patient Register ensures a complete follow-up, allowing for the best possible evaluation of the patient’s medical history before arrest. Second, it is important to consider that OHCA reporting might have been less complete before 2016, and there has been a transition to electronic forms of data collection in 2015–2016. This transition likely improved the accuracy and completeness of data collection, contributing to the apparent increase in reported incidences and number of survivors. Third, to study a more homogenous group of patients we divided OHCAs in this study into presumed cardiac cause vs. non-cardiac causes based on post-treatment diagnosis. Thus, while acknowledging the potential limitations of post-treatment classification, the use of the same definition throughout the study period minimizes bias and allows for meaningful analysis of temporal trends within the study population. Sex-based disparities in various aspects of OHCA management, including medication administration, airway management, and post-resuscitation care, may contribute to observed differences in 30-day survival between men and women. We did not have information on administered medication and other variables related to the resuscitation process for the whole study period. However, previous studies have mainly pointed to sex differences in provision of PCI or CAG after OHCA,^61^ with women receiving less invasive procedures. Therefore, we believe the inclusion of the PCI on the day of OHCA optimize the model. Importantly, our study focused on short-term outcomes of OHCA patients but did not assess neurological recovery. Future research should explore long-term functional outcomes, including return to work and daily assistance needs to better understand the impact of the advances in treatment of OHCA. Lastly, the Danish healthcare system differs from some countries, potentially limiting the generalizability of our results. Sex disparities in outcomes may vary more significantly in other countries, especially where sex differences in lifestyle and access to healthcare systems are more pronounced.^62^ Due to the observational nature of this study, the associations are suggestive rather than indicative of causal effects. Thus, conclusions drawn on causality should be approached with caution.

## Conclusions

Despite increases in 30-day survival rates for both sexes, women consistently had lower survival rates than men, even in adjusted analysis. The largest sex-related differences were found among patients aged 50–75 years, those who were witnessed by a bystander or EMS, and received CPR. In contrast, sex difference diminished in patients with a shockable heart rhythm and being within the Utstein comparator group. No significant difference was found in patients <50 years. Further research is needed to explain why women are not experiencing the same increase in survival as men.

## Supplementary Material

oeaf047_Supplementary_Data

## Data Availability

The data underlying this article cannot be shared publicly due to the privacy of individuals that participated in the study.

## Lead author biography

Nertila Zylyftari is a medical doctor at Svendborg Hospital and affiliated researcher at Gentofte Hospital, Copenhagen, with collaborations at Hillerød Hospital. Her work focuses on preventing out-of-hospital cardiac arrest, particularly early warning signs, symptom patterns, and sex-related differences. Her research has been supported by the European Union's Horizon 2020 Research and Innovation programme under acronym ESCAPE-NET, registered under grant agreement no. 733381, and the COST Action PARQ (grant agreement no. CA19137) supported by COST (European Cooperation in Science and Technology). The ‘European Sudden Cardiac Arrest Network: Towards Prevention, Education, and New Effective Treatments (ESCAPE-NET)' consortium is an international research collaboration with the main aims of improving the knowledge on sudden cardiac arrest and increasing the survival after it. She have also received funding from Helsefonden.
