# Supplementary material for: Sex differences in out-of-hospital cardiac arrest
Source: Eur Heart J Open. 2025 Apr 28;5(3):oeaf047. doi: 10.1093/ehjopen/oeaf047 (PMC12089750; doi:10.1093/ehjopen/oeaf047)

**SUPPLEMENTAL MATERIAL**

**Table S1.** Patient characteristics according to sex and age groups.

| **Characterisitics** | **age≥75 years** | | | | | |  | **age 50-75 years** | |  | **age ≤50 years** |  |
| --- | --- | --- | --- | --- | --- | --- | --- | --- | --- | --- | --- | --- |
|  | **Women**  **(N=8,801)** | | | | | **Men**  **(N=12,211)** | | **Women (N=7,067)** | **Men (N=17,679)** | | **Women (N=1,245)** | **Men**  **(N=3,063)** |
| **Education levels** |  | | | | |  | |  |  | |  |  |
| Basic education | 6,362 (72.3) | | | | | 6,529 (53.5) | | 4,026 (57.0) | 7,078 (40.0) | | 574 (46.1) | 1,431 (46.7) |
| General or vocational upper secondary | 1,707 (19.4) | | | | | 4,047 (33.1) | | 2,191 (31.0) | 7,832 (44.3) | | 468 (37.6) | 1,216 (39.7) |
| Bachelor, Master or Doctoral Degree or equivalent. | 732 (8.3) | | | | | 1,635 (13.4) | | 850 (12.0) | 2,769 (15.7) | | 203 (16.3) | 416 (13.6) |
| H**ousehold i**ncome levels |  | | | | |  | |  |  | |  |  |
| Low | 2,500 (28.4) | | | | | 3,259 (26.7) | | 1,495 (21.2) | 3,119 (17.6) | | 332 (26.7) | 909 (29.7) |
| Medium | 5,090 (57.8) | | | | | 6,515 (53.4) | | 3,610 (51.1) | 8,058 (45.6) | | 633 (50.8) | 1,335 (43.6) |
| High | 1,211 (13.8) | | | | | 2,437 (20.0) | | 1,962 (27.8) | 6,502 (36.8) | | 280 (22.5) | 819 (26.7) |
| GP contact 30 days before OHCA | 5,765 (65.3) | | | | | 7,427 (60.7) | | 3,827 (53.9) | 8,347 (47.0) | | 545 (43.3) | 1,100 (35.4) |
| **Civil status**  Married  Widow | 2,196 (25.0)  4,997 (56.8) | | | | | 7,477 (61.2)  2,768 (22.7) | | 3,536 (50.0)  1,064 (15.1) | 10,563 (59.7)  897 (5.1) | | 437 (35.1)  20 (1.6) | 1,010 (33.0)  10 (0.3) |
| **Number of persons at home**  One (living alone)  Two | 6,016 (68.4)  2,26 (26.4) | | | | | 3,969 (32.5)  7,474 (61.2) | | 2,485 (35.2)  3,683 (52.1) | 4,571 (25.9)  9,985 (56.5) | | 265 (21.3)  3,693 (52.0) | 884 (28.9)  587 (19.2) |
| Comorbidities | |  | | | |  | |  |  | |  |  |
| Hypertension | 5,280 (60.0) | | | | | 7,541 (61.8) | | 3,558 (50.3) | 9,828 (55.6) | | 363 (29.2) | 944 (30.8) |
| Type 2 Diabetes | 1,193 (13.6) | | | | | 2,010 (16.5) | | 1,169 (16.5) | 3,203 (18.1) | | 98 (7.9) | 244 (8.0) |
| Myocardial infarction | 852 (9.7) | | | | | 1,649 (13.5) | | 476 (6.7) | 1,771 (10.0) | | 28 (2.2) | 127 (4.1) |
| Ischaemic heart disease | 1,795 (20.4) | | | | | 3,748 (30.7) | | 1,014 (14.3) | 4,056 (22.9) | | 64 (5.1) | 207 (6.8) |
| Heart failure | 1,801 (20.5) | | | | | 3,284 (26.9) | | 957 (13.5) | 3,048 (17.2) | | 65 (5.2) | 231 (7.5) |
| Atrial fibrillation | 2,113 (24.0) | | | | | 3,628 (29.7) | | 773 (10.9) | 2,569 (14.5) | | 26 (2.1) | 117 (3.8) |
| Stroke | 1,194 (13.6) | | | | | 1,810 (14.8) | | 586 (8.3) | 1,690 (9.6) | | 31 (2.5) | 66 (2.2) |
| Chronic obstructive pulmonary disease (COPD) | 1,691 (19.2) | | | | | 2,317 (19.0) | | 1,532 (21.7) | 2,155 (12.2) | | 61 (4.9) | 80 (2.6) |
| Neurological diesease | 1,905 (21.6) | | | | | 2,000 (16.4) | | 1,464 (20.7) | 3,005 (17.0) | | 329 (26.4) | 712 (23.2) |
| Psychiatric disorders | 865 (9.8) | | | | | 611 (5.0) | | 1,013 (14.3) | 1,336 (7.6) | | 322 (25.9) | 524 (17.1) |
| Drug and alcohol abuse 180 days before OHCA | 286 (3.2) | | | | | 505 (4.1) | | 868 (12.3) | 2,093 (11.8) | | 212 (17.0) | 556 (18.2) |
| Pre-arrest invasive in-hospital procedures (10 years beforeOHCA) | | |  | |  | | |  |  | |  |  |
| Coronary artery bypass graft surgery (CABG) | 78 (0.9) | | | | | 427 (3.5) | | 89 (1.3) | 662 (3.7) | | 5 (0.4) | 21 (0.7) |
| Percutaneous coronary intervention (PCI) | 345 (3.9) | | | | | 849 (7.0) | | 261 (3.7) | 1,243 (7.0) | | 17 (1.4) | 118 (3.9) |
| Pacemaker implantation | 506 (5.7) | | | | | 991 (8.1) | | 172 (2.4) | 676 (3.8) | | 21 (1.7) | 53 (1.7) |
| Coronary angiography (CAG) | 919 (10.4) | | | | | 2,204 (18.0) | | 797 (11.3) | 3,080 (17.4) | | 71 (5.7) | 251 (8.2) |
| **Post-arrest invasive procedures (up to 30 days after OHCA)** | | | |  | |  | |  |  | |  |  |
| PCI | 111 (1.3) | | | | | 322 (2.6) | | 261 (3.7) | 1,652 (9.3) | | 79 (6.3) | 294 (9.6) |
| CAG | 219 (2.5) | | | | | 784 (6.4) | | 648 (9.2) | 3,081 (17.4) | | 239 (19.2) | 662 (21.6) |
| **Medication 180 days before and at the time of OHCA** | | | |  | |  | |  |  | |  |  |
| Beta blocker, calcium antagonist, or digoxin | 4,567 (51.9) | | | | | 6,308 (51.7) | | 2,656 (37.6) | 7,112 (40.2) | | 164 (13.2) | 459 (15.0) |
| Antidepressant or anti-psychotic drugs | 2,920 (33.2) | | | | | 2,465 (20.2) | | 2,314 (32.7) | 3,110 (17.6) | | 377 (30.3) | 597 (19.5) |
| Anticoagulant drugs | 1,537 (17.5) | | | | | 3,014 (24.7) | | 709 (10.0) | 2,391 (13.5) | | 37 (3.0) | 120 (3.9) |
| Systemic steroids | 1,864 (21.2) | | | | | 2,396 (19.6) | | 1,690 (23.9) | 2,397 (13.6) | | 121 (9.7) | 172 (5.6) |
| Medication related to COPD | 2,117 (24.1) | | | | | 2,909 (23.8) | | 2,126 (30.1) | 3,000 (17.0) | | 185 (14.9) | 248 (8.1) |
| Antianginal drugs (Ivabradine, nitrate, nicorandil, nitroglycerin) | 1,020 (11.6) | | | | | 1,745 (14.3) | | 444 (6.3) | 1,417 (8.0) | | 19 (1.5) | 56 (1.8) |
| Statins | 2,233 (25.4) | | | | | 4,203 (34.4) | | 2,005 (28.4) | 5,882 (33.3) | | 89 (7.1) | 314 (10.3) |
| Antibiotics 30 days before and at the time of OHCA | 1,994 (22.7) | | | | | 2,246 (18.4) | | 1,332 (18.8) | 2,049 (11.6) | | 162 (13.0) | 221 (7.2) |
| QT-prolonging drugs 30 days before and at the time of OHCA | 1,285 (14.6) | | | | | 1,250 (10.2) | | 994 (14.1) | 1,437 (8.1) | | 139 (11.2) | 204 (6.7) |
| **Cardiac arrest related factors** | | | |  | |  | |  |  | |  |  |
| Arrest in private home  Missing (N) | 6,163 (83.7)  1,438 | | | | | 7,918 (80.6)  2,391 | | 4,629 (84.0)  1,558 | 9,762 (71.4)  4,000 | | 738 (77.4)  291 | 1,468 (62.5)  713 |
| Witnessed arrest  Missing (N) | 4,326 (49.6)  78 | | | | | 6,309 (52.1)  96 | | 3,232 (46.0)  49 | 9,449 (53.9)  137 | | 617 (49.9)  8 | 1,522 (50.3)  38 |
| Cardiopulmonary resuscitation before ambulance arrival  Missing (N) | 5,000 (57.3)  74 | | | | | 6,442 (53.0)  65 | | 4,027 (57.3)  37 | 10,612 (60.4)  105 | | 832 (67.0)  4 | 1,996 (65.6)  22 |
| Defibrillation before ambulance arrival  Missing (N) | 286 (3.4)  294 | | | | | 571 (4.8)  285 | | 246 (3.6)  178 | 1,164 (6.8)  457 | | 59 (4.9)  30 | 227 (7.7)  114 |
| Median response time in minutes* (IQR)  Missing (N) | 9 [6, 14]  788 | | | | | 10 [6, 15]  966 | | 9 [6, 15]  566 | 10 [6, 15]  1,411 | | 9 [5, 15]  99 | 10 [6, 15]  280 |
| Initial shockable heart rhythm  Missing (N) | 827 (9.9)  477 | | | | | 2,311 (19.9)  578 | | 1,045 (15.5)  339 | 5,388 (31.8)  728 | | 344 (28.9)  57 | 997 (34.1)  143 |
| Patient has ROSC or has a GCS > 8 at the hospital arrival  Missing (N) | 1,117 (13.3)  395 | | | | | 1,810 (15.3)  411 | | 1,451 (21.3)  239 | 4,179 (24.5)  636 | | 404 (33.6)  42 | 831 (28.4)  132 |

| ***** Interval time from the recognition of OHCA to the first rhythm analysis by Emergency Medical Services.  ROSC, Return of spontaneous circulation |  |  |  |
| --- | --- | --- | --- |

**Table S2.** Patient characteristics according to sex and on weather was bystander witnessed or not. After exclusions of patients with missing data, N= 49,662.

| **Characterisitics** | **Non bystander witnessed OHCAs** | | | | | |  | **Bystander witnessed OHCAs** | | |  |
| --- | --- | --- | --- | --- | --- | --- | --- | --- | --- | --- | --- |
|  | **Women (N=8,804)** | | | **Men (N=15,403)** | | | | **Women (N=8,175)** | | **Men**  **(N=17,280)** | |
| **Age (years), median (IQR)** | 75 [65, 84] | | | | 71 [61, 80] | | | | 76 [66, 85] | 71 [61, 80] | |
| **Age categories** |  | | | |  | | | |  |  | |
| <50 | 620 (7.0) | | | | 1,503 (9.8) | | | | 617 (7.5) | 1,522 (8.8) | |
| 50-75 | 3,787 (43.0) | | | | 8,094 (52.5) | | | | 3,232 (39.5) | 9,449 (54.7) | |
| >75 | 4,397 (49.9) | | | | 5,806 (37.7) | | | | 4,326 (52.9) | 6,309 (36.5) | |
| **Education levels** |  | | | |  | | | |  |  | |
| Basic education | 5,573 (63.3) | | | | 7,344 (47.7) | | | | 5,297 (64.8) | 7,558 (43.7) | |
| General or vocational upper secondary | 2,309 (26.2) | | | | 5,967 (38.7) | | | | 2,024 (24.8) | 7,029 (40.7) | |
| Bachelor, Master or Doctoral Degree or equivalent. | 922 (10.5) | | | | 2,092 (13.6) | | | | 854 (10.4) | 2,693 (15.6) | |
| H**ousehold i**ncome levels |  | | | |  | | | |  |  | |
| Low | 2,163 (24.6) | | | | 3,589 (23.3) | | | | 2,101 (25.7) | 3,604 (20.9) | |
| Medium | 4,883 (55.5) | | | | 7,678 (49.8) | | | | 4,395 (53.8) | 8,110 (46.9) | |
| High | 1,758 (20.0) | | | | 4,136 (26.9) | | | | 1,679 (20.5) | 5,566 (32.2) | |
| Comorbidities |  | | | |  | | | |  |  | |
| Hypertension | 4,530 (51.5) | | | | 7,894 (51.2) | | | | 4,600 (56.3) | 10,269 (59.4) | |
| Type 2 Diabetes | 1,278 (14.5) | | | | 2,577 (16.7) | | | | 1,162 (14.2) | 2,845 (16.5) | |
| Myocardial infarction | 651 (7.4) | | | | 1,535 (10.0) | | | | 698 (8.5) | 1,981 (11.5) | |
| Ischaemic heart disease | 1,388 (15.8) | | | | 3,588 (23.3) | | | | 1,459 (17.8) | 4,358 (25.2) | |
| Heart failure | 1,361 (15.5) | | | | 2,915 (18.9) | | | | 1,440 (17.6) | 3,594 (20.8) | |
| Atrial fibrillation | 1,424 (16.2) | | | | 2,910 (18.9) | | | | 1,466 (17.9) | 3,346 (19.4) | |
| Stroke | 906 (10.3) | | | | 1,668 (10.8) | | | | 884 (10.8) | 1,869 (10.8) | |
| Chronic obstructive pulmonary disease  (COPD) | 1,735 (19.7) | | | | 2,245 (14.6) | | | | 1,526 (18.7) | 2,270 (13.1) | |
| Neurological diesease | 1,992 (22.6) | | | | 2,985 (19.4) | | | | 1,670 (20.4) | 2,683 (15.5) | |
| Psychiatric disorders | 1,245 (14.1) | | | | 1,326 (8.6) | | | | 938 (11.5) | 1,131 (6.5) | |
| Drug and alcohol abuse 180 days before OHCA | 846 (9.6) | | | | 1,768 (11.5) | | | | 508 (6.2) | 1,353 (7.8) | |
| Pre-arrest invasive in-hospital procedures (10 years before OHCA) | |  | | |  | | | |  |  | |
| Coronary artery bypass graft surgery (CABG) | 69 (0.8) | | | | 475 (3.1) | | | | 100 (1.2) | 629 (3.6) | |
| Percutaneous coronary intervention (PCI) | 314 (3.6) | | | | 926 (6.0) | | | | 305 (3.7) | 1,261 (7.3) | |
| Pacemaker implantation | 332 (3.8) | | | | 803 (5.2) | | | | 361 (4.4) | 902 (5.2) | |
| Coronary angiography (CAG) | 883 (10.0) | | | | 2,390 (15.5) | | | | 894 (10.9) | 3,112 (18.0) | |
| Surgery for cardiac arrhythmias or conduction disorders, including implantable cardioverter-defibrillator  and radiofrequency ablation | 79 (0.9) | | | | 342 (2.2) | | | | 90 (1.1) | 448 (2.6) | |
| **Post-arrest invasive procedures (up to 30 days after OHCA)** | | |  | |  | | | |  |  | |
| PCI | 98 (1.1) | | | | 398 (2.6) | | | | 350 (4.3) | 1,848 (10.7) | |
| CAG | 239 (2.7) | | | | 786 (5.1) | | | | 862 (10.5) | 3,698 (21.4) | |
| **Medication 180 days before OHCA** | | | | | |  | | | | | |
| Beta blocker, calcium antagonist, or digoxin | 3,773 (42.9) | | | | 6,471 (42.0) | | | | 3,554 (43.5) | 7,301 (42.3) | |
| Antidepressant or anti-psychotic drugs | 2,940 (33.4) | | | | 3,115 (20.2) | | | | 2,634 (32.2) | 3,011 (17.4) | |
| Anticoagulant drugs | 1,154 (13.1) | | | | 2,546 (16.5) | | | | 1,114 (13.6) | 2,940 (17.0) | |
| Systemic steroids | 1,879 (21.3) | | | | 2,319 (15.1) | | | | 1,771 (21.7) | 2,613 (15.1) | |
| COPD medication | 2,302 (26.1) | | | | 2,972 (19.3) | | | | 2,100 (25.7) | 3,143 (18.2) | |
| Antianginal drugs (Ivabradine, nitrate, nicorandil, nitroglycerin) | 713 (8.1) | | | | 1,425 (9.3) | | | | 756 (9.2) | 1,760 (10.2) | |
| Statins | 2,217 (25.2) | | | | 4,668 (30.3) | | | | 2,083 (25.5) | 5,664 (32.8) | |
| Antibiotics 30 days before OHCA | 1,739 (19.8) | | | | 2,160 (14.0) | | | | 1,714 (21.0) | 2,332 (13.5) | |
| QT-prolonging drugs 30 days before OHCA | 1,178 (13.4) | | | | 1,385 (9.0) | | | | 1,215 (14.9) | 1,477 (8.5) | |
| **Cardiac arrest related factors** |  | | | |  | | | |  |  | |
| Arrest in private home  Missing (N) | 6,310 (89.0)  1,712 | | | | 9,703 (80.4)  3,340 | | | | 5,148 (77.5)  1,532 | 9,318 (68.5)  3,675 | |
| Cardiopulmonary resuscitation before ambulance arrival  Missing (N) | 4,737 (54.0)  30 | | | | 7,980 (51.9)  28 | | | | 5,089 (62.3)  13 | 10,995 (63.8)  38 | |
| Defibrillation before ambulance arrival  Missing (N) | 223 (2.6)  181 | | | | 504 (3.3)  289 | | | | 367 (4.6)  235 | 1,454 (8.6)  430 | |
| Median response time in minutes* (IQR) | 9 [5, 14] | | | | 9 [6, 15] | | | | 10 [6, 15] | 10 [6, 16] | |
| Missing (N) | 735 | | | | 1,206 | | | | 638 | 1,315 | |
| Initial shockable heart rhythm  Missing (N) | 548 (6.6)  473 | | | | 1,947 (13.3)  729 | | | | 1,659 (21.2)  345 | 6,663 (40.1)  658 | |
| Patient has ROSC or has a GCS > 8 at the hospital arrival  Missing (N) | 806 (9.4)  274 | | | | 1,446 (9.7)  441 | | | | 2,161 (27.5)  319 | 5,347 (32.1)  603 | |

| ***** Interval time from the recognition of OHCA to the first rhythm analysis by Emergency Medical Services.  ROSC, Return of spontaneous circulation |  |  |  |
| --- | --- | --- | --- |

**Table S3.** Patient characteristics according to sex and on weather was performed or not cardiopulmonary resuscitation. After exclusions of patients with missing data, N= 49,760.

| **Characterisitics** | **OHCAs with no CPR** | | | | |  | **OHCAs with CPR** | |  |
| --- | --- | --- | --- | --- | --- | --- | --- | --- | --- |
|  | **Women**  **(N=7,140)** | | **Men**  **(N=13,711)** | | | | **Women (N=9,859)** | **Men**  **(N=19,050)** | |
| **Age (years), median (IQR)** | 76 [66, 84] | | 73 [64, 81] | | | | 76 [65, 85] | 70 [60, 79] | |
| **Age categories** |  | |  | | | |  |  | |
| <50 | 409 (5.7) | | 1,045 (7.6) | | | | 832 (8.4) | 1,996 (10.5) | |
| 50-75 | 3,004 (42.1) | | 6,962 (50.8) | | | | 4,027 (40.8) | 10,612 (55.7) | |
| >75 | 3,727 (52.2) | | 5,704 (41.6) | | | | 5,000 (50.7) | 6,442 (33.8) | |
| **Education levels** |  | |  | | | |  |  | |
| Basic education | 4,930 (69.0) | | 6,876 (50.1) | | | | 5,953 (60.4) | 8,074 (42.4) | |
| General or vocational upper secondary | 1,613 (22.6) | | 5,167 (37.7) | | | | 2,729 (27.7) | 7,850 (41.2) | |
| Bachelor, Master or Doctoral Degree or equivalent. | 597 (8.4) | | 1,668 (12.2) | | | | 1,177 (11.9) | 3,126 (16.4) | |
| H**ousehold i**ncome levels |  | |  | | | |  |  | |
| Low | 2,808 (39.3) | | 4,553 (33.2) | | | | 1,465 (14.9) | 2,670 (14.0) | |
| Medium | 3,329 (46.6) | | 6,376 (46.5) | | | | 5,954 (60.4) | 9,450 (49.6) | |
| High | 1,003 (14.0) | | 2,782 (20.3) | | | | 2,440 (24.7) | 6,930 (36.4) | |
| Comorbidities |  | |  | | | |  |  | |
| Hypertension | 3,795 (53.2) | | 7,346 (53.6) | | | | 5,352 (54.3) | 10,855 (57.0) | |
| Type 2 Diabetes | 1,018 (14.3) | | 2,188 (16.0) | | | | 1,434 (14.5) | 3,248 (17.0) | |
| Myocardial infarction | 655 (9.2) | | 1,691 (12.3) | | | | 693 (7.0) | 1,837 (9.6) | |
| Ischaemic heart disease | 1,256 (17.6) | | 3,675 (26.8) | | | | 1,595 (16.2) | 4,292 (22.5) | |
| Heart failure | 1,254 (17.6) | | 2,960 (21.6) | | | | 1,546 (15.7) | 3,567 (18.7) | |
| Atrial fibrillation | 1,135 (15.9) | | 2,622 (19.1) | | | | 1,757 (17.8) | 3,660 (19.2) | |
| Stroke | 734 (10.3) | | 1,619 (11.8) | | | | 1,055 (10.7) | 1,930 (10.1) | |
| Chronic obstructive pulmonary disease (COPD) | 1,349 (18.9) | | 2,091 (15.3) | | | | 1,919 (19.5) | 2,438 (12.8) | |
| Neurological diesease | 1,332 (18.7) | | 2,132 (15.5) | | | | 2,337 (23.7) | 3,549 (18.6) | |
| Psychiatric disorders | 822 (11.5) | | 906 (6.6) | | | | 1,364 (13.8) | 1,555 (8.2) | |
| Drug or alcohol abuse 180 days before OHCA | 527 (7.4) | | 1,255 (9.2) | | | | 830 (8.4) | 1,872 (9.8) | |
| Pre-arrest invasive in-hospital procedures  (10 years before OHCA) | | | |  |  | |  |  | |
| Coronary artery bypass graft surgery (CABG) | 73 (1.0) | | 488 (3.6) | | | | 96 (1.0) | 617 (3.2) | |
| Percutaneous coronary intervention (PCI) | 234 (3.3) | | 797 (5.8) | | | | 386 (3.9) | 1,401 (7.4) | |
| Pacemaker implantation | 220 (3.1) | | 665 (4.9) | | | | 472 (4.8) | 1,045 (5.5) | |
| Coronary angiography (CAG) | 655 (9.2) | | 2,065 (15.1) | | | | 1,127 (11.4) | 3,450 (18.1) | |
| Surgery for cardiac arrhythmias or conduction disorders, including implantable cardioverter-defibrillator and radiofrequency ablation | 48 (0.7) | | 237 (1.7) | | | | 121 (1.2) | 558 (2.9) | |
| **Post-arrest invasive procedures (up to 30 days after OHCA)** | |  |  | | | |  |  | |
| PCI | 126 (1.8) | | 427 (3.1) | | | | 322 (3.3) | 1,821 (9.6) | |
| CAG | 257 (3.6) | | 824 (6.0) | | | | 844 (8.6) | 3,664 (19.2) | |
| **Medication 180 days before and at the time of OHCA** |  | |  | | | |  |  | |
| Beta blocker, calcium antagonist, or digoxin | 3,128 (43.8) | | 6,087 (44.4) | | | | 4,205 (42.7) | 7,720 (40.5) | |
| Antidepressant or anti-psychotic drugs | 2,166 (30.3) | | 2,432 (17.7) | | | | 3,413 (34.6) | 3,704 (19.4) | |
| Anticoagulant drugs | 769 (10.8) | | 2,078 (15.2) | | | | 1,504 (15.3) | 3,424 (18.0) | |
| Systemic steroids | 1,504 (21.1) | | 2,177 (15.9) | | | | 2,152 (21.8) | 2,765 (14.5) | |
| COPD medication | 1,787 (25.0) | | 2,761 (20.1) | | | | 2,620 (26.6) | 3,369 (17.7) | |
| Antianginal drugs (Ivabradine, nitrate, nicorandil, nitroglycerin) | 711 (10.0) | | 1,662 (12.1) | | | | 761 (7.7) | 1,541 (8.1) | |
| Statins | 1,606 (22.5) | | 3,941 (28.7) | | | | 2,691 (27.3) | 6,420 (33.7) | |
| Antibiotics 30 days before and at the time of OHCA | 1,348 (18.9) | | 1,883 (13.7) | | | | 2,115 (21.5) | 2,619 (13.7) | |
| QT-prolonging drugs 30 days before and at the time of OHCA | 984 (13.8) | | 1,280 (9.3) | | | | 1,415 (14.4) | 1,592 (8.4) | |
| **Cardiac arrest related factors** |  | |  | | | |  |  | |
| Arrest in private home  Missing (N) | 4,024 (88.1)  2,573 | | 7,323 (85.1)  5,103 | | | | 7,441 (81.1)  688 | 11,716 (68.6)  1,966 | |
| Wtnessed arrest  Missing (N) | 3,073 (43.2)  30 | | 6,247 (45.8)  69 | | | | 5,089 (51.8)  33 | 10,995 (57.9)  76 | |
| Defibrillation before ambulance arrival  Missing (N) | 27 (0.4)  234 | | 73 (0.5)  359 | | | | 564 (5.8)  186 | 1,885 (10.1)  370 | |
| Median response time in minutes* (IQR)  Missing (N) | 10 [7, 17]  532 | | 11 [7, 16]  900 | | | | 8 [5, 13]  843 | 9 [6, 14]  1,630 | |
| Initial shockable heart rhythm  Missing (N) | 756 (11.2)  412 | | 2,714 (20.8)  688 | | | | 1,448 (15.3)  421 | 5,921 (32.2)  721 | |
| Patient has ROSC or has a GCS > 8 at the hospital arrival  Missing (N) | 833 (12.2)  339 | | 1,643 (12.5)  526 | | | | 2,136 (22.2)  258 | 5,160 (27.9)  526 | |

| ***** Interval time from the recognition of OHCA to the first rhythm analysis by Emergency Medical Services.  ROSC, Return of spontaneous circulation |  |  |  |
| --- | --- | --- | --- |

**Table S4.** Patient characteristics according to sex and initial shockable heart rhythm. After exclusions of patients with missing data, N= 47,760.

| **Characterisitics** | **OHCAs with non-shockable rhythm** | | | | | |  | | **OHCAs with shockable rhythm** | |  |
| --- | --- | --- | --- | --- | --- | --- | --- | --- | --- | --- | --- |
|  | **Women (N=14,033)** | | | | | **Men**  **(N=22,817)** | | | **Women (N=2,214)** | **Men**  **(N=8,696)** | |
| Age (years), median (median, IQR) | 77 [67, 85] | | | | | 72 [63, 81] | | | 70 [58, 81] | 68 [58, 76] | |
| **Age categories** |  | | | | |  | | |  |  | |
| <50 | 845 (6.0) | | | | | 1,927 (8.4) | | | 344 (15.5) | 997 (11.5) | |
| 50-75 | 5,685 (40.5) | | | | | 11,566 (50.7) | | | 1,043 (47.1) | 5,388 (62.0) | |
| >75 | 7,503 (53.5) | | | | | 9,324 (40.9) | | | 827 (37.4) | 2,311 (26.6) | |
| **Education levels** |  | | | | |  | | |  |  | |
| Basic education | 9,091 (64.8) | | | | | 10,881 (47.7) | | | 1,307 (59.0) | 3,447 (39.6) | |
| General or vocational upper secondary | 3,503 (25.0) | | | | | 8,821 (38.7) | | | 638 (28.8) | 3,750 (43.1) | |
| Bachelor, Master or Doctoral Degree or equivalent. | 1,439 (10.3) | | | | | 3,115 (13.7) | | | 269 (12.1) | 1,499 (17.2) | |
| H**ousehold i**ncome levels |  | | | | |  | | |  |  | |
| Low | 3,466 (24.7) | | | | | 5,191 (22.8) | | | 611 (27.6) | 1,701 (19.6) | |
| Medium | 7,813 (55.7) | | | | | 11,365 (49.8) | | | 1,058 (47.8) | 3,849 (44.3) | |
| High | 2,754 (19.6) | | | | | 6,261 (27.4) | | | 545 (24.6) | 3,146 (36.2) | |
| Comorbidities |  | | | | |  | | |  |  | |
| Hypertension | 7,260 (51.7) | | | | | 11,762 (51.5) | | | 1,486 (67.1) | 5,774 (66.4) | |
| Type 2 Diabetes | 2,033 (14.5) | | | | | 4,000 (17.5) | | | 306 (13.8) | 1,238 (14.2) | |
| Myocardial infarction | 1,023 (7.3) | | | | | 2,326 (10.2) | | | 260 (11.7) | 1,072 (12.3) | |
| Ischaemic heart disease | 2,230 (15.9) | | | | | 5,301 (23.2) | | | 497 (22.4) | 2,379 (27.4) | |
| Heart failure | 2,200 (15.7) | | | | | 4,432 (19.4) | | | 471 (21.3) | 1,848 (21.3) | |
| Atrial fibrillation/flutter | 2,325 (16.6) | | | | | 4,411 (19.3) | | | 448 (20.2) | 1,622 (18.7) | |
| Stroke | 1,490 (10.6) | | | | | 2,642 (11.6) | | | 223 (10.1) | 758 (8.7) | |
| Chronic obstructive pulmonary disease | 2,883 (20.5) | | | | | 3,738 (16.4) | | | 233 (10.5) | 603 (6.9) | |
| Neurological diesease | 3,217 (22.9) | | | | | 4,712 (20.7) | | | 286 (12.9) | 755 (8.7) | |
| Psychiatric disorders | 1,900 (13.5) | | | | | 2,026 (8.9) | | | 197 (8.9) | 353 (4.1) | |
| Drug or alcohol abuse 180 days before OHCA | 1,170 (8.3) | | | | | 2,579 (11.3) | | | 121 (5.5) | 440 (5.1) | |
| Pre-arrest invasive in-hospital procedures (10 years before OHCA) | | |  | |  | | |  | |  | |
| Coronary artery bypass graft surgery (CABG) | 112 (0.8) | | | 703 (3.1) | | | | 54 (2.4) | | 370 (4.3) | |
| Percutaneous coronary intervention (PCI) | 478 (3.4) | | | 1,393 (6.1) | | | | 113 (5.1) | | 722 (8.3) | |
| Pacemaker implantation | 537 (3.8) | | | 1,172 (5.1) | | | | 133 (6.0) | | 486 (5.6) | |
| Coronary angiography (CAG) | 1,373 (9.8) | | | 3,590 (15.7) | | | | 337 (15.2) | | 1,733 (19.9) | |
| Surgery for cardiac arrhythmias or conduction disorders, including implantable cardioverter-defibrillator and radiofrequency ablation | 124 (0.9) | | | 575 (2.5) | | | | 37 (1.7) | | 184 (2.1) | |
| **Post-arrest invasive procedures (up to 30 days after OHCA)** | |  | |  | | | |  | |  | |
| PCI | 99 (0.7) | | | 496 (2.2) | | | | 343 (15.5) | | 1,724 (19.8) | |
| CAG | 343 (2.4) | | | 1,174 (5.1) | | | | 738 (33.3) | | 3,240 (37.3) | |
| Medication 180 days before OHCA |  | | |  | | | |  | |  | |
| Beta blocker, calcium antagonist, or digoxin | 6,033 (43.0) | | | 9,586 (42.0) | | | | 984 (44.4) | | 3,701 (42.6) | |
| Antidepressant or anti-psychotic drugs | 4,848 (34.5) | | | 5,002 (21.9) | | | | 459 (20.7) | | 864 (9.9) | |
| Anticoagulant drugs | 1,843 (13.1) | | | 3,815 (16.7) | | | | 333 (15.0) | | 1,487 (17.1) | |
| Systemic steroids | 3,215 (22.9) | | | 3,950 (17.3) | | | | 261 (11.8) | | 773 (8.9) | |
| COPD medication | 3,855 (27.5) | | | 4,920 (21.6) | | | | 354 (16.0) | | 955 (11.0) | |
| Antianginal drugs (Ivabradine, nitrate, nicorandil, nitroglycerin) | 1,148 (8.2) | | | 2,167 (9.5) | | | | 254 (11.5) | | 903 (10.4) | |
| Statins | 3,534 (25.1) | | | 6,993 (30.5) | | | | 610 (27.5) | | 3,031 (34.8) | |
| Antibiotics 30 days before and at the time of OHCA | 2,996 (21.3) | | | 3,600 (15.8) | | | | 314 (14.2) | | 716 (8.2) | |
| QT-prolonging drugs 30 days before and at the time of OHCA | 2,079 (14.8) | | | 2,318 (10.2) | | | | 213 (9.6) | | 429 (4.9) | |
| **Cardiac arrest related factors** |  | | |  | | | |  | |  | |
| Arrest in private home  Missing (N) | 9,796 (84.9)  2,525 | | | 14,366 (78.7)  4,558 | | | | 1,263 (73.0)  485 | | 4,088 (61.0)  1,996 | |
| Witnessed arrest  Missing (N) | 6,174 (44.2)  73 | | | 9,963 (43.9)  123 | | | | 1,659 (75.2)  7 | | 6,663 (77.4)  86 | |
| Cardiopulmonary resuscitation before ambulance arrival  Missing (N) | 7,995 (57.3)  65 | | | 12,412 (54.6)  91 | | | | 1,448 (65.7)  10 | | 5,921 (68.6)  61 | |
| Defibrillation before ambulance arrival  Missing (N) | 431 (3.1)  343 | | | 1,115 (5.0)  513 | | | | 142 (6.6)  67 | | 783 (9.3)  236 | |
| Median response time in minutes* (IQR)  Missing (N) | 9 [5, 14]  1,125 | | | 9 [6, 15]  1,785 | | | | 11 [6, 16]  157 | | 11 [7, 16]  593 | |
| Patient has ROSC or has a GCS > 8 at the hospital arrival  Missing (N) | 1,880 (13.9)  487 | | | 2,925 (13.2)  709 | | | | 1,011 (47.6)  90 | | 3,733 (44.7)  340 | |

| ***** Interval time from the recognition of OHCA to the first rhythm analysis by Emergency Medical Services.  ROSC, Return of spontaneous circulation |  |  |  |
| --- | --- | --- | --- |

**Table S5.** Patient characteristics among EMS-witnessed OHCAs by sex, N=6,392.

| **Characterisitics** | **Women**  **(n=2,302)** | | | | | **Men**  **(n=4,090)** |
| --- | --- | --- | --- | --- | --- | --- |
| Age (years) (IQR) | 76 [66, 84] | | | | | 70 [60, 79] |
| Ed**ucation levels** |  | | | | |  |
| Basic education | 1,470 (63.9) | | | | | 1,837 (44.9) |
| General or vocational upper secondary | 578 (25.1) | | | | | 1,655 (40.5) |
| Bachelor, Master or Doctoral Degree or equivalent | 254 (11.0) | | | | | 598 (14.6) |
| H**ousehold i**ncome levels |  | | | | |  |
| Low | 598 (26.0) | | | | | 858 (21.0) |
| Medium | 1,232 (53.5) | | | | | 2,042 (49.9) |
| High | 472 (20.5) | | | | | 1,190 (29.1) |
| Comorbidities | |  | | | |  |
| Hypertension | 1,380 (59.9) | | | | | 2,588 (63.3) |
| Type 2 Diabetes | 387 (16.8) | | | | | 717 (17.5) |
| Myocardial infarction | 221 (9.6) | | | | | 490 (12.0) |
| Ischaemic heart disease | 449 (19.5) | | | | | 977 (23.9) |
| Heart failure | 377 (16.4) | | | | | 737 (18.0) |
| Atrial fibrillation/flutter | 398 (17.3) | | | | | 635 (15.5) |
| Stroke | 264 (11.5) | | | | | 438 (10.7) |
| Chronic obstructive pulmonary disease (COPD) | 392 (17.0) | | | | | 512 (12.5) |
| Neurological dieseases | 428 (18.6) | | | | | 701 (17.1) |
| Psychiatric disorders | 241 (10.5) | | | | | 310 (7.6) |
| Drug or alcohol abuse 180 days before OHCA | 134 (5.8) | | | | | 401 (9.8) |
| Pre-arrest invasive in-hospital procedures (10 years before OHCA) | | | | |  |  |
| Coronary artery bypass graft surgery (CABG) | 27 (1.2) | | | | | 126 (3.1) |
| Percutaneous coronary intervention (PCI) | 109 (4.7) | | | | | 341 (8.3) |
| Pacemaker | 81 (3.5) | | | | | 189 (4.6) |
| Coronary angiography (CAG) | 268 (11.6) | | | | | 687 (16.8) |
| Surgery for cardiac arrhythmias or conduction disorders, including implantable cardioverter-defibrillator and radiofrequency ablation | 22 (1.0) | | | | | 106 (2.6) |
| **Post-arrest invasive procedures (up to 30 days after OHCA)** | | | |  | |  |
| PCI | 264 (11.5) | | | | | 900 (22.0) |
| KAG | 371 (16.1) | | | | | 1,176 (28.8) |
| **Medication 180 days before and at the time of OHCA** | | |  | | |  |
| Beta blocker, calcium antagonist, or digoxin | 1,060 (46.0) | | | | | 1,681 (41.1) |
| Antidepressant or anti-psychotic drugs | 684 (29.7) | | | | | 764 (18.7) |
| Anticoagulant drugs | 257 (11.2) | | | | | 535 (13.1) |
| Systemic steroider | 433 (18.8) | | | | | 657 (16.1) |
| COPD medication | 543 (23.6) | | | | | 762 (18.6) |
| Antianginal drugs (Ivabradine, nitrate, nicorandil, nitroglycerin) | 237 (10.3) | | | | | 404 (9.8) |
| Statins | 625 (27.1) | | | | | 1,278 (31.2) |
| Antibiotics 30 days before and at the time of OHCA | 463 (20.1) | | | | | 628 (15.4) |
| QT-prolonging drugs 30 days before and at the time of OHCA | 278 (12.1) | | | | | 382 (9.3) |
| **Cardiac arrest related factors** |  | | | | |  |
| Arrest in private home  Missing (N) | 1,142 (61.4)  442 | | | | | 1,916 (59.0)  848 |
| Initial shockable heart rhythm  Missing (N) | 309 (14.2)  129 | | | | | 864 (22.2)  209 |
| Patient has ROSC or has a GCS > 8 at the hospital arrival  Missing (N) | 764 (34.7)  107 | | | | | 1,615(41.3)  184 |
| ROSC, Return of spontaneous circulation |  | | | | |  |

**Table S6.** Patient characteristics according to sex of Utstein population (non-traumatic, bystander witnessed OHCA presenting with an initial shockable heart rhythm), N= 8322.

| **Characterisitics** | **Women (N=1,659)** | | | | **Men (N=6,663)** |
| --- | --- | --- | --- | --- | --- |
| Age (years) (IQR) | 70 [57, 81] | | | | 67 [58, 76] |
| **Education levels** |  | | | |  |
| Basic education | 990 (59.7) | | | | 2,597 (39.0) |
| General or vocational upper secondary | 464 (28.0) | | | | 2,886 (43.3) |
| Bachelor, Master or Doctoral Degree or equivalent. | 205 (12.4) | | | | 1,180 (17.7) |
| H**ousehold i**ncome levels |  | | | |  |
| Low | 455 (27.4) | | | | 1,269 (19.0) |
| Medium | 795 (47.9) | | | | 2,900 (43.5) |
| High | 409 (24.7) | | | | 2,494 (37.4) |
| Comorbidities |  | | | |  |
| Hypertension | 1,145 (69.0) | | | | 4,520 (67.8) |
| Type 2 Diabetes | 229 (13.8) | | | | 948 (14.2) |
| Myocardial infarction | 202 (12.2) | | | | 830 (12.5) |
| Ischaemic heart disease | 377 (22.7) | | | | 1,806 (27.1) |
| Heart failure | 358 (21.6) | | | | 1,429 (21.4) |
| Atrial fibrillation/flutter | 336 (20.3) | | | | 1,204 (18.1) |
| Stroke | 154 (9.3) | | | | 571 (8.6) |
| Chronic obstructive pulmonary disease (COPD) | 172 (10.4) | | | | 436 (6.5) |
| Neurological diesease | 196 (11.8) | | | | 565 (8.5) |
| Psychiatric disorders | 144 (8.7) | | | | 277 (4.2) |
| Drug or alcohol abuse 180 days before OHCA | 80 (4.8) | | | | 326 (4.9) |
| Pre-arrest invasive in-hospital procedures (10 years before OHCA) | | | |  |  |
| Coronary artery bypass graft surgery (CABG) | 41 (2.5) | | | | 293 (4.4) |
| Percutaneous coronary intervention (PCI) | 84 (5.1) | | | | 560 (8.4) |
| Pacemaker | 97 (5.8) | | | | 341 (5.1) |
| Coronary angiography (CAG) | 255 (15.4) | | | | 1,336 (20.1) |
| Surgery for cardiac arrhythmias or conduction disorders, including implantable cardioverter-defibrillator and radiofrequency ablation | 28 (1.7) | | | | 148 (2.2) |
| **Post-arrest invasive procedures (up to 30 days after OHCA)** | | |  | |  |
| PCI | 270 (16.3) | | | | 1,409 (21.1) |
| KAG | 597 (36.0) | | | | 2,683 (40.3) |
| **Medication 180 days before and at the time of OHCA** | |  | | |  |
| Beta blocker, calcium antagonist, or digoxin | 726 (43.8) | | | | 2,818 (42.3) |
| Antidepressant or anti-psychotic drugs | 329 (19.8) | | | | 668 (10.0) |
| Anticoagulant drugs | 258 (15.6) | | | | 1,117 (16.8) |
| Systemic steroider | 209 (12.6) | | | | 561 (8.4) |
| COPD medication | 271 (16.3) | | | | 711 (10.7) |
| Antianginal drugs (Ivabradine, nitrate, nicorandil, nitroglycerin) | 183 (11.0) | | | | 702 (10.5) |
| Statins | 450 (27.1) | | | | 2,321 (34.8) |
| Antibiotics 30 days before and at the time of OHCA | 234 (14.1) | | | | 552 (8.3) |
| QT-prolonging drugs 30 days before and at the time of OHCA | 158 (9.5) | | | | 330 (5.0) |
| **Cardiac arrest related factors** |  | | | |  |
| Private home  Missing (N) | 924 (72.0)  375 | | | | 3,050 (59.5)  1536 |
| Cardiopulmonary resuscitation before ambulance arrival** | 1,129 (68.2) | | | | 4,718(71.0) |
| Defibrillation before ambulance arrival  Missing (N) | 107(6.7)  54 | | | | 622 (9.6)  167 |
| Median response time in minutes* (IQR)  Missing (N) | 11 [6, 17]  117 | | | | 11 [6, 17]  448 |
| Patient has ROSC or has a GCS > 8 at the hospital arrival  Missing (N) | 828 (52.3)  75 | | | | 3,107 (48.4)  250 |

| ***** Interval time from the recognition of OHCA to the first rhythm analysis by Emergency Medical Services.  ** Missing not shown as they were < 4.  ROSC, Return of spontaneous circulation |  |  |  |
| --- | --- | --- | --- |

**Table S7.** The association between 30-day survival and the covariates included in the adjusted logistic regression model presented in Figure 4. These estimates are adjusted with all covariates in the model. Odds ratio of 30-day survival by sex of OHCA population in men as compared to women (reference). CPR, Cardiopulmonary resuscitation; PCI, Percutaneous coronary intervention.

|  | **survival 30 d** | | |
| --- | --- | --- | --- |
| *Predictors* | *Odds Ratios* | *CI* | *p* |
| Intercept | 0.03 | 0.02 – 0.03 | **<0.0001** |
| Males | 1.29 | 1.15 – 1.45 | **<0.0001** |
| Age 50-75 | 0.45 | 0.39 – 0.53 | **<0.0001** |
| Age >75 | 0.20 | 0.17 – 0.24 | **<0.0001** |
| Respiratory Disease | 0.68 | 0.61 – 0.77 | **<0.0001** |
| Hypertension | 0.84 | 0.74 – 0.95 | **0.0066** |
| Ischamisc heart disease | 1.13 | 0.98 – 1.31 | 0.1042 |
| Heart Failure | 0.96 | 0.82 – 1.12 | 0.5669 |
| Type 2 Diabetes | 0.62 | 0.54 – 0.73 | **<0.0001** |
| Antidepressant and antipsychiatric drugs | 0.73 | 0.63 – 0.84 | **<0.0001** |
| Antibiotics within 30 days | 0.69 | 0.58 – 0.82 | **<0.0001** |
| QT-prolonging drugs within 30 days | 0.82 | 0.66 – 1.02 | 0.0798 |
| Private home | 0.47 | 0.42 – 0.53 | **<0.0001** |
| Witnessed arrest | 1.83 | 1.63 – 2.07 | **<0.0001** |
| CPR | 1.34 | 1.17 – 1.54 | **<0.0001** |
| Shockable heart rhythm | 2.91 | 2.60 – 3.25 | **<0.0001** |
| Defibrillation | 1.92 | 1.65 – 2.25 | **<0.0001** |
| Response time >10 min | 0.78 | 0.70 – 0.87 | **<0.0001** |
| Patient has ROSC or has a GCS > 8 at the hospital arrival | 45.48 | 39.35 – 52.79 | **<0.0001** |
| PCI after OHCA | 1.66 | 1.44 – 1.91 | **<0.0001** |
| Observations | 28417 | | |
| R^2^ Tjur | 0.523 | | |

**Figure S1.** Flowchart of study population. Abbreviations: OHCA, Out-of-hospital cardiac arrest; EMS, Emergency Medical Services.

**
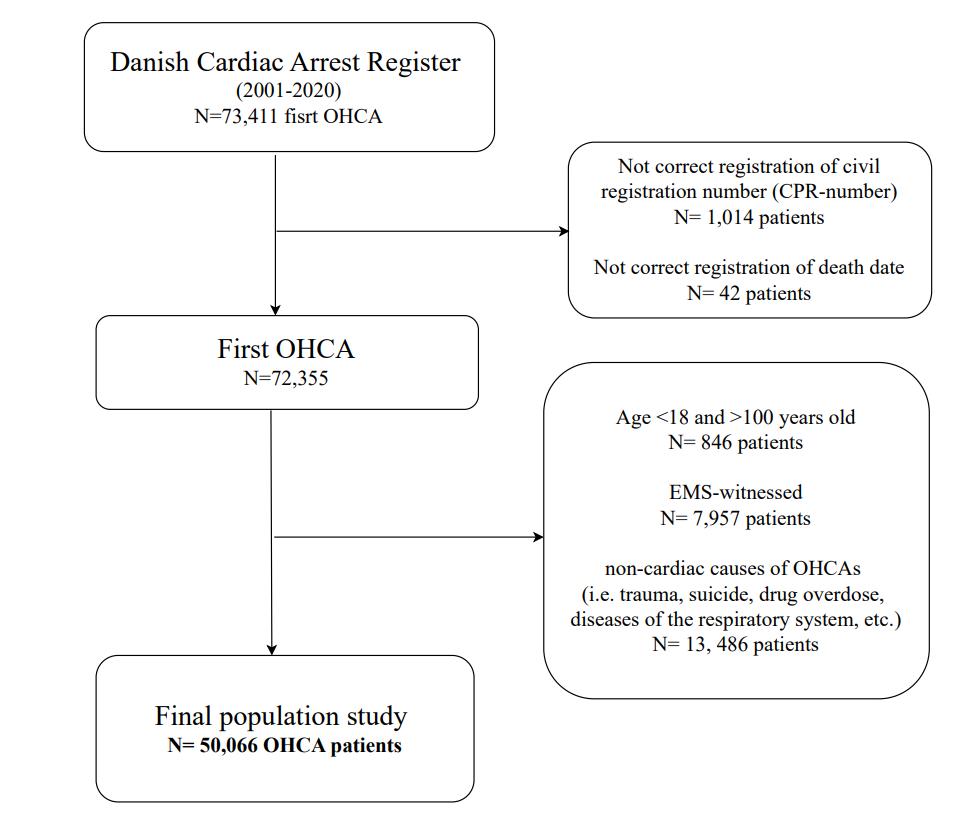
**

**Figure S2.** The incidence and the 30-day survival in absolute numbers among OHCA patients by sex. From 2015 to 2016, the numbers increased drastically for both sexes, due to a transition to electronic forms in data collection.


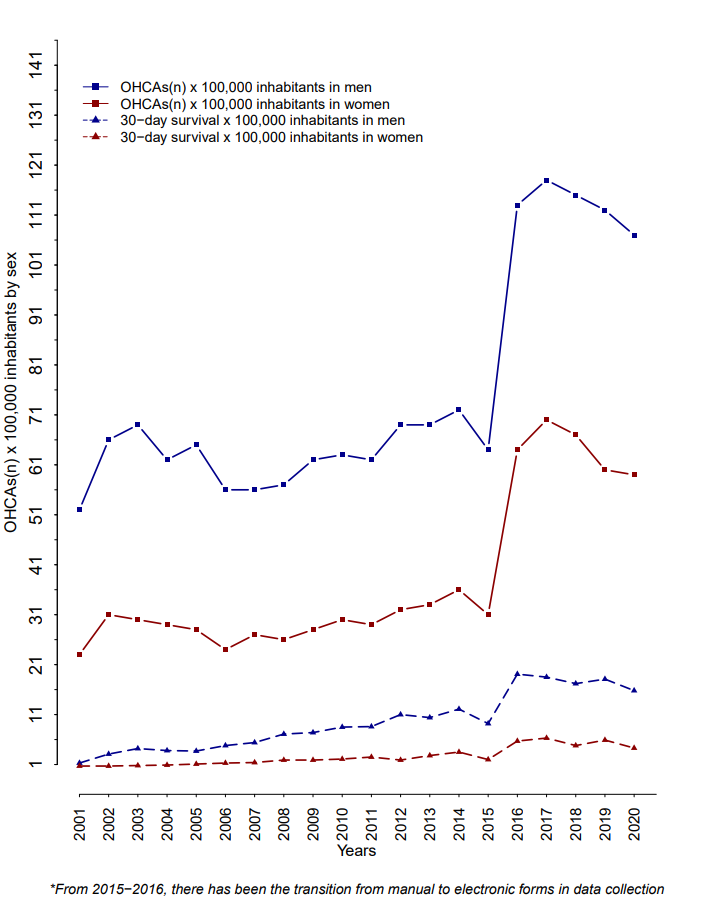


**Figure S3**. The absolute number of survivors among men and women during the study period.


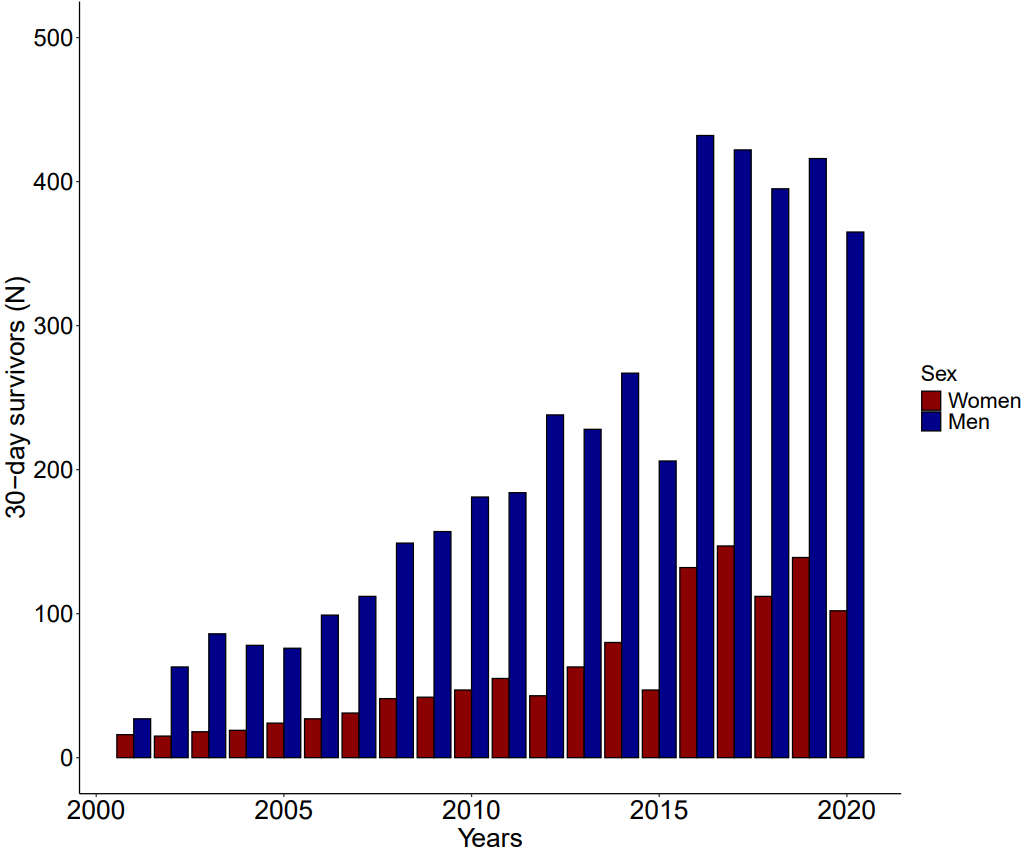


**Figure S4.** Temporal trends of cardiac arrest related factors in the study population during a 20-year period. Men vs Women with bystander-witnessed; Men vs Women with CPR; Men vs Women with initial shockable heart rhythm; Men vs Women with ROSC. CPR, Cardiopulmonary resuscitation; ROSC, Return of spontaneous circulation.


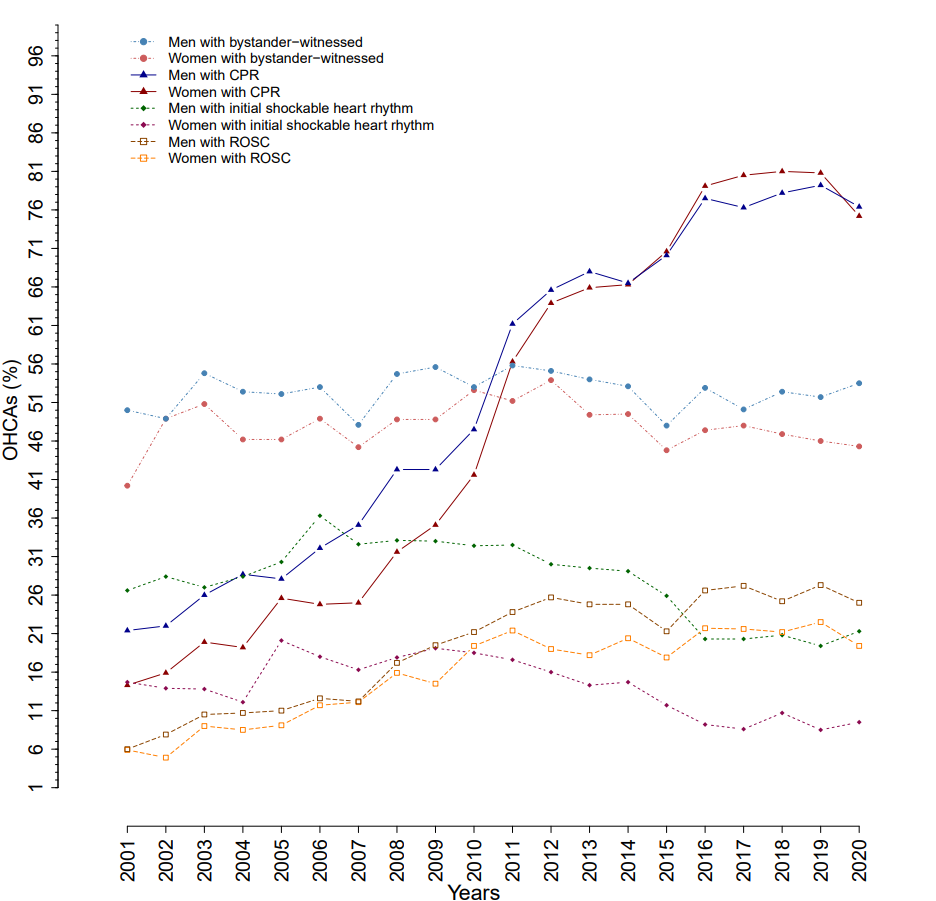


**Figure S5.** OHCA incidence (n x 100,000 inhabitants per year) and crude 30-day survival (in percentages) by sex from 2001-2020 among OHCA patients witnessed by EMS.


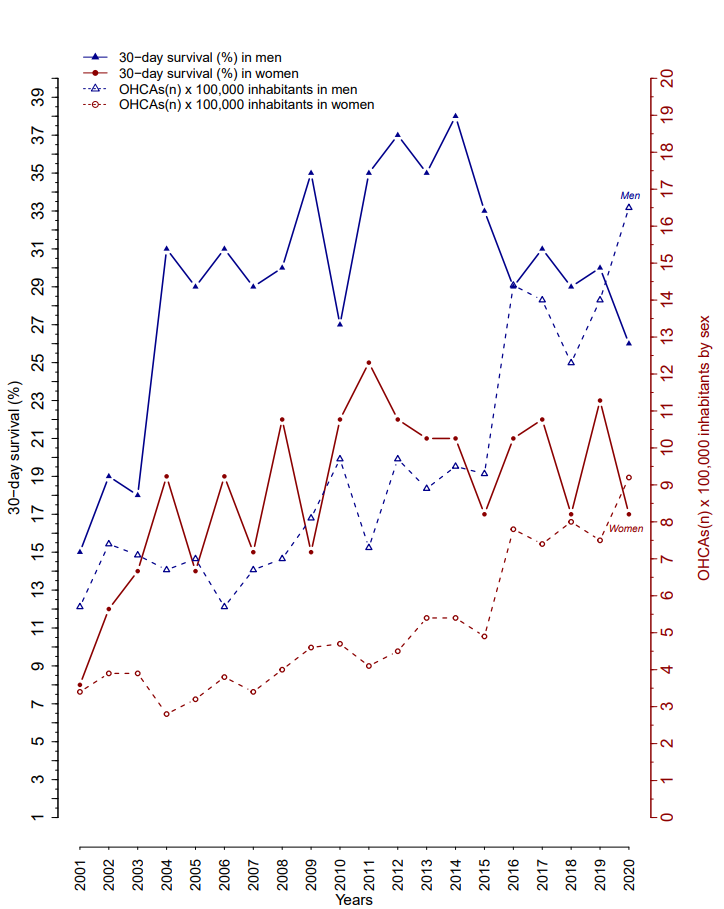


**Figure S6.** OHCA incidence (n x 100,000 inhabitants per year) and crude 30-day survival (in percentages by sex from 2001-2020 among Utstein population (non-traumatic, bystander witnessed OHCA presenting with an initial shockable heart rhythm).


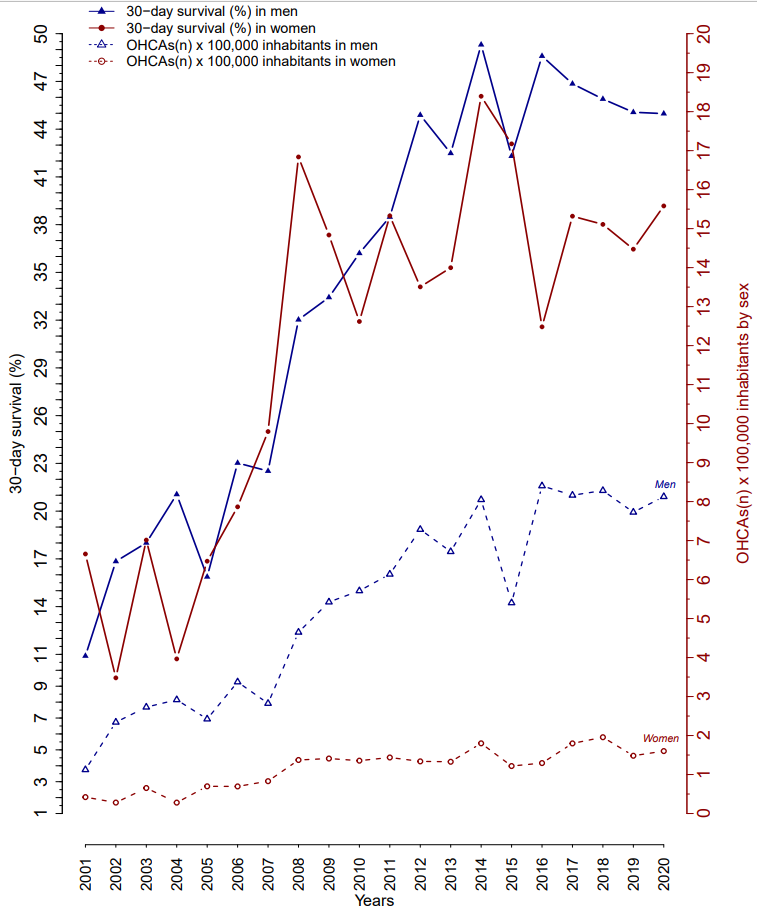

Supplement: oeaf047_Supplementary_Data [file oeaf047_supplementary_data.docx]
